# Impact of Multisensor CIED-based Heart Failure Monitoring on Mortality, Heart Failure Hospitalizations and Outpatient Visits: A Systematic Review

**DOI:** 10.1007/s11897-025-00707-y

**Published:** 2025-07-01

**Authors:** Bert A. C. Zwaenepoel, Annefleur Kluft, Michelle Feijen, Jan W. Schoones, Ward A. Heggermont, Anastasia D. Egorova, Saskia L.M.A. Beeres

**Affiliations:** 1 Department of Cardiology, AZ Delta, Roeselare, Belgium; 2 Department of Cardiology, Leiden University Medical Centre, Leiden, The Netherlands; 3 Directorate of Research Policy, Leiden University Medical Center, Leiden, the Netherlands; 4 Cardiovascular Research Centre Aalst, Department of Cardiology, OLV Clinic, Aalst, Belgium

**Keywords:** Heart Failure, Remote Monitoring, Cardiac Implantable Electronic Devices (CIEDs), HeartLogic, TriageHF, HeartInsight

## Abstract

**Purpose of Review:**

Cardiac Implantable Electronic Device (CIED)-based remote monitoring has been proposed to improve heart failure (HF) management by enabling early detection of decompensation. This systematic review evaluates the effectiveness of multisensor CIED-based monitoring in reducing mortality, HF hospitalizations, and unplanned HF outpatient visits.

**Recent Findings:**

Earlier CIED-based remote monitoring strategies were mainly based on single-sensor impedance-based algorithms, and showed limited clinical benefits. Newer multisensor CIED-based algorithms have shown promise in initial studies. However, their impact on clinical outcomes remains uncertain, and therefore current HF guidelines provide limited recommendations.

**Summary:**

Multisensor CIED-based algorithms reliably identify high-risk HF patients and their use leads to reductions in HF hospitalizations and unplanned outpatient HF visits, although prospective validation in RCTs is lacking for any of the algorithms. Standardized response strategies are needed to enhance clinical integration and generalizability. If validated, multisensor monitoring could become a key tool in HF management.

**Supplementary Information:**

The online version contains supplementary material available at 10.1007/s11897-025-00707-y.

## Introduction

Heart failure (HF) is a complex clinical syndrome that afflicts more than 60 million people worldwide, corresponding to a prevalence of 1–2% of adults [1]. It is associated with high morbidity and mortality and is characterized by disease progression with episodes of sudden clinical deterioration. These episodes frequently lead to hospitalizations which impair patients’ prognosis and pose a significant burden on healthcare resources [2]. As the general population ages and the overall survival after the diagnosis improves due to advances in treatment strategies, the prevalence of HF and the number of hospitalizations is projected to increase in the forthcoming years [1].

For these reasons, prevention of (recurrent) hospitalizations is one of the major goals in HF management [3]. Timely detection of impending fluid retention is crucial in order to create a window for timely therapeutic adjustments in the ambulant setting and thereby avoid hospitalizations. In the last decades, remote monitoring of parameters reflecting objective measures of worsening HF has gained interest as a promising solution to detect impeding decompensation at an early stage.

**Fig. 1:**
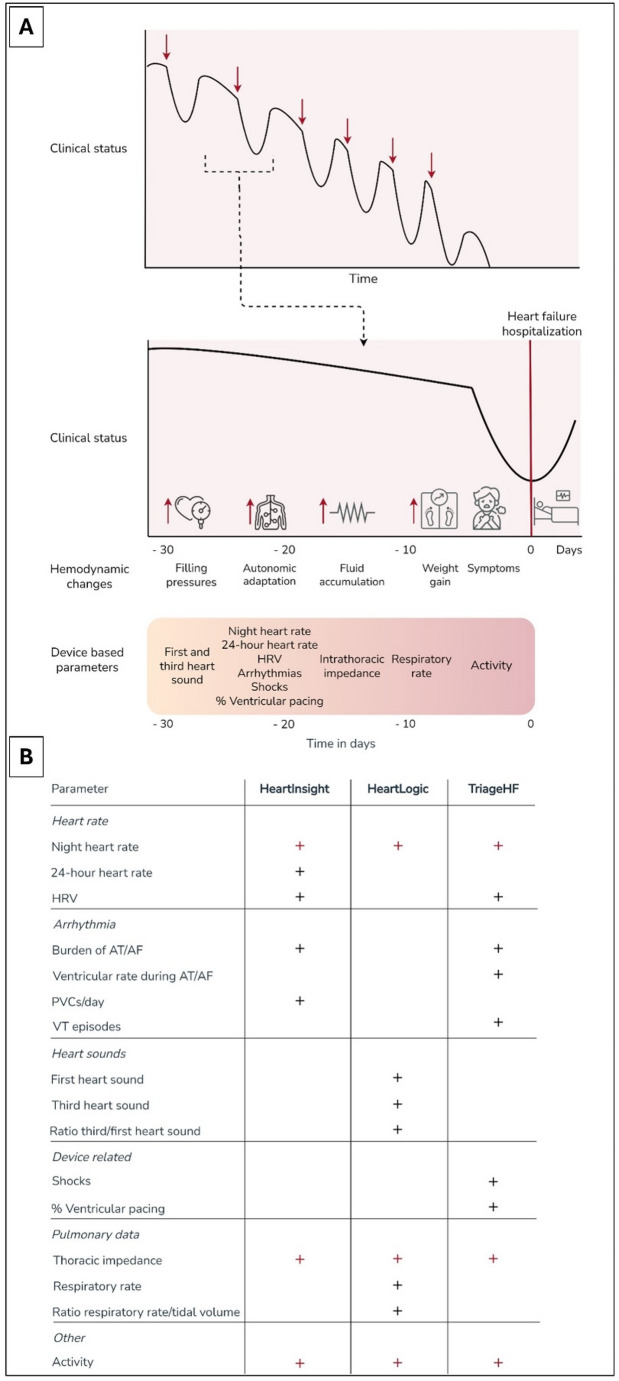
The chronic heart failure path and its physiological changes detectable by CIEDs ***Panel A***: *Traditional perspective on the progression of chronic heart failure and the early physiological changes detectable by contemporary cardiac implantable electronic device (CIED) algorithms prior to the onset of decompensation related symptoms.**** Panel B***: *schematic representation of the specific parameters integrated into the commercially available algorithms HeartInsight*,* HeartLogic*,* and TriageHF.*

The first remote monitoring strategies consisted of assessments of body weight, heart rate and blood pressure at home and monitoring of symptoms through telephone contact. Such strategies showed significant reductions in all-cause mortality and HF hospitalizations [4–6]. Simultaneously, Cardiac Implantable Electronic Devices (CIEDs) have established an important role in the treatment of HF to prevent sudden cardiac death and/or improve cardiac function by means of resynchronization [7, 8]. The sensors built-in in these devices have facilitated a potential next step in remote monitoring. Sensing of electrical resistance, heart frequency and acceleration allow for monitoring of parameters such as heart sounds, thoracic impedance, respiratory rate, heart rate variability, night heart rate and physical activity (Fig. 1A).

These parameters have all been described to correlate with the pathophysiology process of worsening HF [9–16]. However, their individual ability to predict an upcoming episode of worsening HF in clinical practice is limited [13, 17, 18]. Two previous meta-analyses were unable to demonstrate a beneficial effect of primarily impedance-driven, CIED-based monitoring on HF hospitalizations and all-cause mortality [19, 20]. Single sensor impedance-driven monitoring fails to provide the solution the field is seeking for. As a result, the current ESC guidelines only give a class IIB, level of evidence B, recommendation for the use of non-invasive home remote monitoring for HF patients to reduce the risk of hospitalizations and death, while no recommendations are available specifically for CIED-based remote monitoring [3].

The limited success of single sensor monitoring strategies may be attributed to the inherent multifactorial and complex nature of the pathophysiology of worsening HF. For this reason, the predictive value of integrating multiple parameters was further investigated and several CIED manufactures developed integrated multisensor algorithms to detect impeding decompensation. These models are developed by Biotronik SE & Co. KG (Berlin, Germany), Boston Scientific (Marlborough, Massachusetts, USA) and Medtronic (Minneapolis, Minnesota, USA) and named HeartInsight, HeartLogic and TriageHF, respectively (Fig. 1B). The initial validation studies showed promising results [21–23]. In recent years, with advancements further accelerated by the COVID-19 pandemic, studies evaluating the feasibility and utility of these multisensor CIED-based algorithms to guide HF management have been published. To date, whether such multisensor strategies (either commercially available or custom made) can be used to guide HF management and improve clinical outcomes has not been systematically evaluated. One previously published meta-analysis focusing on HF management guided by multiparametric monitoring did not focus on the inclusion of recent studies with commercially available algorithms, which are now increasingly used in practice and bridge an important gap in the facilitation of clinical implementation [24]. Furthermore, urgent outpatient HF visits have not been systematically evaluated in the context of multisensory monitoring. Therefore, this review addresses this gap by encompassing data on both customized parameter configurations and commercially available CIED algorithms, while specifically addressing the individual endpoints of mortality, HF hospitalization and urgent outpatient HF visits as a primary outcome.

## Methods

A systematic review encompassing randomized controlled trials (RCTs) and observational studies, adhering to the Preferred Reporting Items for Systematic Reviews and Meta-Analyses (PRISMA) guidelines was conducted. A comprehensive literature search spanning from January 2012 to July 2024, utilizing databases including PubMed, Embase, Web of Science, Emcare, and the Cochrane Library was performed. The search terms used were ‘heart failure’, ‘cardiac implantable electronic device’, ‘cardiac resynchronization therapy’, ‘implantable cardioverter defibrillator’, ‘multisensor’, ‘mortality’, ‘hospitalizations’, ‘outpatient visit’ and ‘remote monitoring’. The detailed search strategy was made by an independent librarian and is provided in Supplemental Table 1. The search strategy exclusively included published, peer-reviewed original articles written in English. This study was registered in the PROSPERO database under the registration number CRD42024565990. Inclusion criteria encompassed studies featuring multisensor CIED-based HF home monitoring and exploring their capability to discern patients at risk of mortality, HF hospitalizations, and/or unplanned outpatient HF visits for heart failure requiring dose escalation of loop diuretics, as well as assessing the potential risk reduction in these clinical outcomes associated with the use of these algorithms. Whenever specific numbers for HF hospitalizations were not available, cardiovascular (CV) hospitalizations were used as a proxy. Multisensor CIED-based HF home monitoring was defined as remote follow-up incorporating at least two distinct parameters, measured by a CIED. These algorithms were either customized or commercially available (HeartInsight, HeartLogic and TriageHF). For customized parameter sets, each included study defined a specific combination of CIED-based parameters to be monitored, but were required to include at least one of the following parameters to be included in the present analysis: thoracic impedance, patient activity, and/or nocturnal heart rate. These parameters are key indicators for detecting early signs of HF decompensation and are consistently integrated into all three commercially available algorithms (Fig. 1B). For studies with customized parameter designs, if it was unclear which parameters were remotely monitored, an email inquiry to the corresponding author was performed. Studies were excluded if no response was received. We excluded editorials, (systematic) reviews and meta-analyses.

Two reviewers (B.Z. and A.K.) independently conducted title and abstract screening to identify studies potentially meeting the inclusion criteria. Discrepancies in eligibility were resolved through consensus. Subsequently, the full texts of identified studies were independently retrieved and scrutinized by the two reviewers. Each study was meticulously deliberated to determine eligibility based on predefined inclusion and exclusion criteria. In instances of unresolved disagreement, final adjudication was done by two additional reviewers (S.B. and A.E.). Two reviewers (B.Z. and A.K.) independently collected data from the selected reports on a standardized manner. The process included independent review and confirmation by additional reviewers (S.B. and A.E.) in cases of discrepancy. No automation tools were used in the data extraction or confirmation process. No publicly available data collection forms, extracted data, analytic code, or other materials were used in this review. All data were independently extracted from the included studies and analyzed using internal resources specific to this study.

In cases where eligible studies examined the same or an overlapping cohort, preference was given to the study with the longest follow-up duration or the most recent publication covering the entire cohort, unless differentiating outcomes were investigated in each article.

To assess the risk of bias of each study included, the quality assessment tool “QUALSYST” from the “Standard Quality Assessment Criteria for Evaluating Primary Research Papers from a Variety of Fields” was used (Supplemental Table 2) [25]. Using this tool, 14 items from each quantitative study were assessed at both the study and outcome levels, with scores assigned based on the extent to which specific criteria were met or reported: “yes” = 2, “partial” = 1, and “no” = 0. Items deemed not applicable to a particular study design were marked as “n/a” and excluded from the calculation of the overall summary score.

For the meta-analysis of data available on reduction of mortality, HF hospitalizations, and/or unplanned outpatient HF visits, a random-effects model with inverse-variance weighting was applied using the meta package in R (Rstudio version 2024.04.2). The analysis pooled logarithmic incidence rate ratios (IRRs) and standard errors derived from the studies. Effect sizes were presented as IRRs with 95% confidence intervals (95% CI) for the outcome of interest. Forest plots were generated using the meta package in R. For sensitivity analysis purposes, results from a fixed-effects model were also displayed. A two-sided p-value of < 0.05 was considered statistically significant. Heterogeneity was tested using the I²-statistic and Cochran’s Q-test, based on a restricted maximum-likelihood estimator.

## Results

### Study Characteristics

**Fig. 2:**
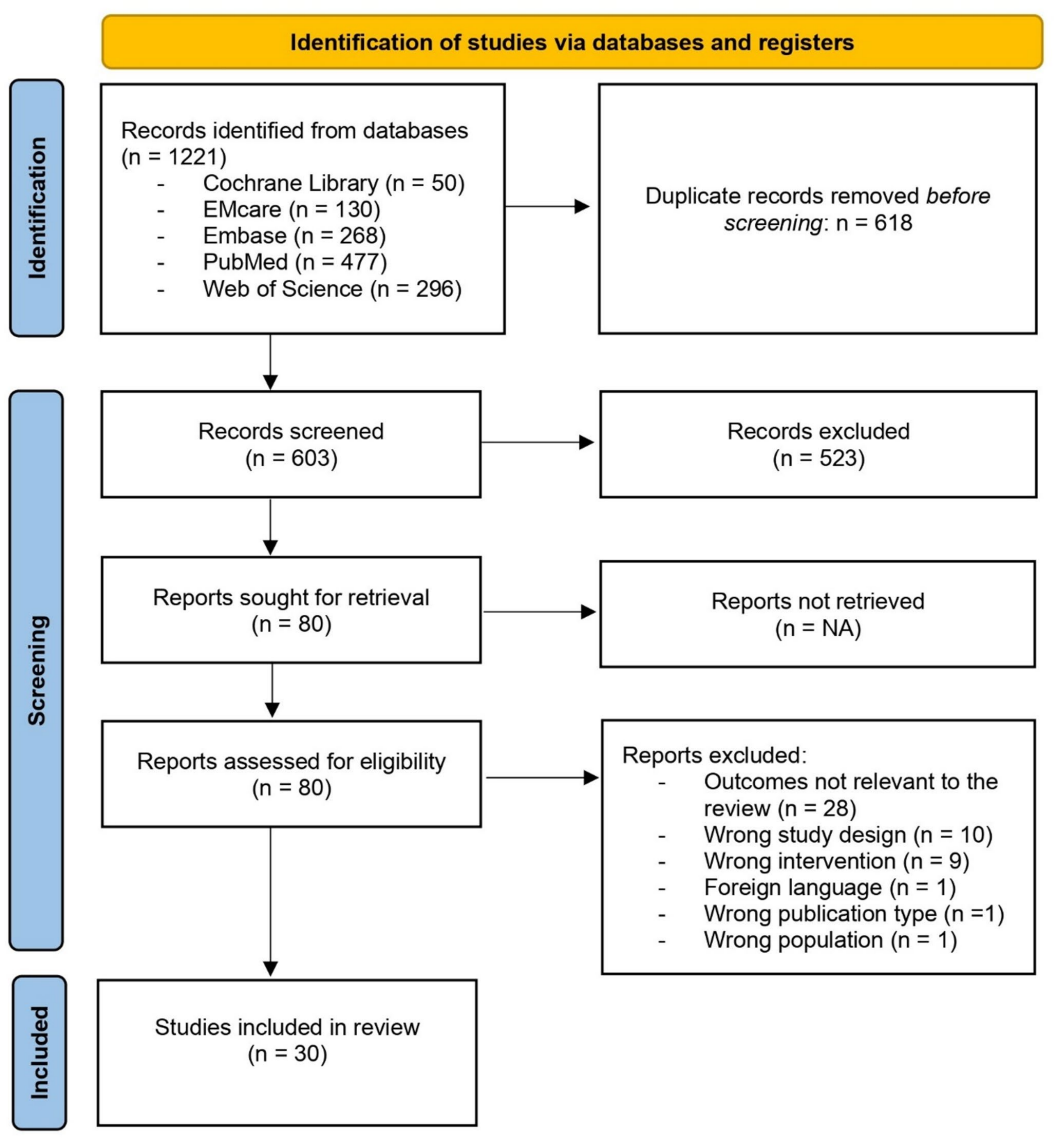
PRISMA flow diagram *PRISMA flow diagram illustrating the selection process for studies included in the systematic review. The diagram details the number of records identified*,* screened*,* assessed for eligibility*,* and included*,* along with reasons for exclusion at each stage*

Up to July 3th 2024, 603 unique articles were identified. After screening title and abstract, 80 articles were selected. After assessing the full-text, 30 articles were selected for the systematic review. The full PRISMA flow diagram is shown in Fig. 2.

The main characteristics (first author, journal, year of publication, type of study, parameter(s) used, number of included patients, median age, gender, etiology of heart failure, New York Heart Association (NYHA) class, mean left ventricular ejection fraction (LVEF), follow-up duration, intervention, comparator (if applicable), main outcomes (primary and/or secondary), and results of the 30 selected articles are shown in Supplemental Table 3. Study sample sizes ranged from 40 to 22901 patients.

**Fig. 3:**
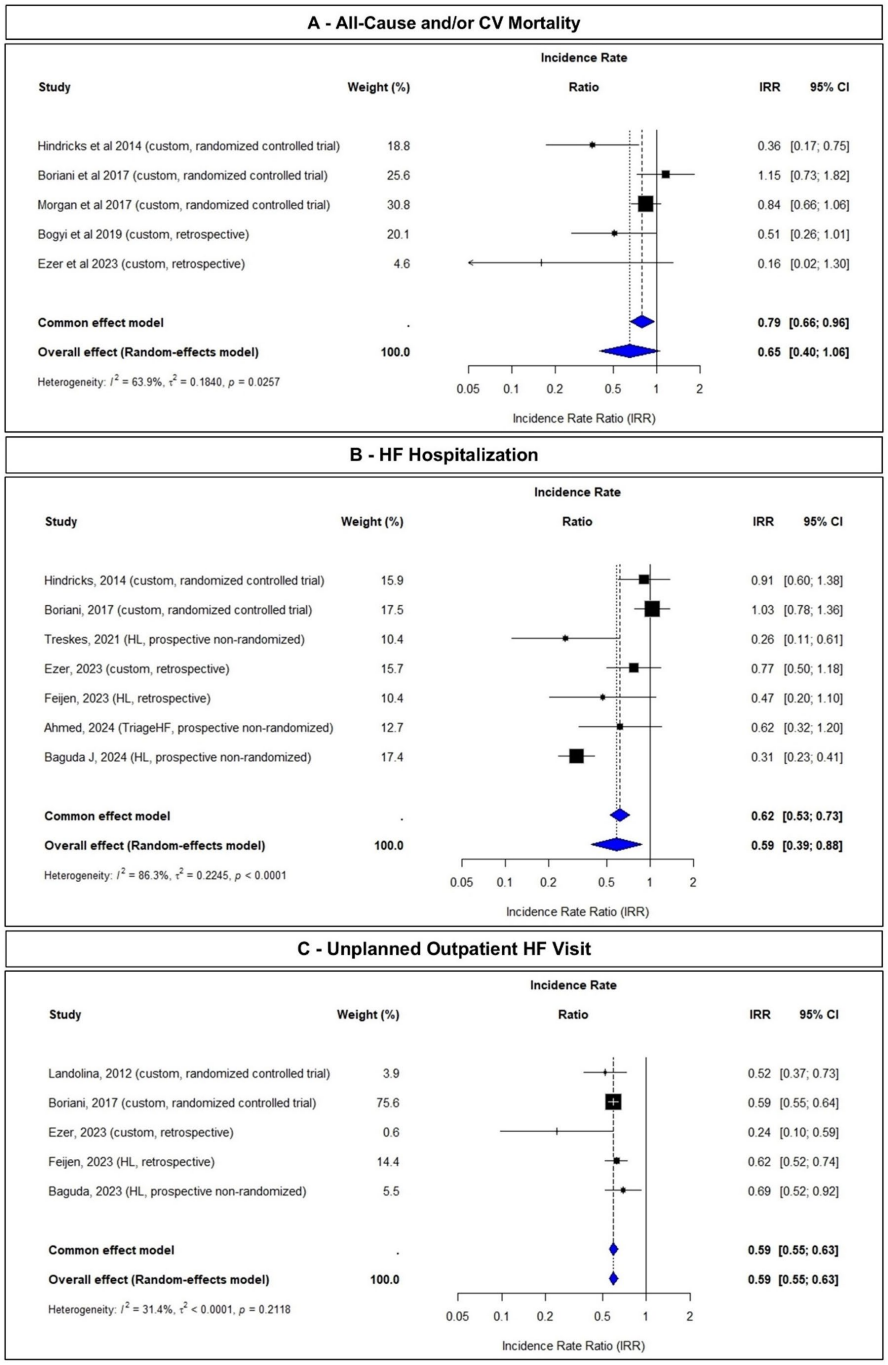
Comparison of clinical outcomes in multisensor CIED-based HF monitoring: all-cause or cardiovascular mortality, heart failure hospitalization, and urgent outpatient heart failure visits ***Panel A***: *studies reporting all-cause and/or cardiovascular mortality as primary outcomes.* *** Panel B***: *studies focusing on heart failure hospitalization as an outcome.* ***Panel C***: *studies evaluating unplanned outpatient heart failure visits as an outcome*

Table 1 summarizes the main data of the selected studies on multisensory CIED-based algorithms identifying patients at risk of: (A) all-cause and/or cardiovascular mortality, (B) heart failure hospitalizations, and/or (C) unplanned outpatient HF visits for heart failure. In contrast, outcome data of the included studies evaluating the ability of multisensory CIED-based algorithms to effectively reduce the prevalence of the aforementioned endpoints are presented in Fig. 3.

**Table 1:** Available literature on the ability of CIED-based multisensor algorithms to discern patients at high risk for all-cause/cardiovascular mortality, heart failure hospitalization and unplanned outpatient heart failure visits

First author, year	Multiparametric model	Primary or secondary outcome	Result
**QUESTION 1. ALL-CAUSE AND/OR CARDIOVASCULAR MORTALITY**			
Gardner R. 2018.	HeartLogic	All-cause mortality (primary, though part of a composite endpoint)	ERR 7.05 (4.69–10.61, *P* < 0.0001)
Zile M. 2020.	TriageHF	All-cause mortality (secondary) - Low vs. intermediate HFRS - Low vs. high HFRS	aHR 1.8 (1.4–2.2, *P* < 0.001) aHR 3.5 (2.8–4.3, *P* < 0.001)
Calo L. 2021.	HeartLogic	CV mortality (primary, though part of a composite endpoint)	HR 30.63 (13.04–71.95) *P* < 0.001
Ahmed F. 2022.	TriageHF	All-cause mortality (primary) - High vs. ‘never-high’ HFRS	OR 3.07 (1.57–6.58, *P* = 0.002)
Baguda J. 2022.	HeartLogic	CV mortality (primary, though part of a composite endpoint)	Sensitivity 98%, specificity 90% PPV 29%, NPV 99.9%
D’Onofrio A. 2022.	HeartInsight	CV mortality (secondary, though part of a composite endpoint)	Sensitivity 54.8%, specificity 86.5%
D’Onofrio A. 2023.	HeartLogic	All-cause mortality (primary)	IRR 13.72 (7.62–25.60, *P* < 0.001)
Santobuono V. 2023.	HeartLogic	All-cause mortality (secondary, though part of a composite endpoint)	IRR 13.35 (8.83–20.51) *P* < 0.001
**QUESTION 2. HF-RELATED HOSPITALIZATIONS**			
Cowie M. 2013.	TriageHF	HF hospitalization (primary) - Low vs. intermediate HFRS - Low vs. high HFRS	HR 2.1 (1.3–3.4, P 0.001) HR 10.0 (6.4–15.7, *P* < 0.001)
Gula L. 2014.	TriageHF	HF hospitalization (primary) - Low vs. intermediate HFRS - Low vs. high HFRS	RR 2.9 (2.0–4.4) RR 10.7 (6.9–16.6)
Sharma V. 2015.	Customized	HF hospitalization (primary) − 0 vs. 1 device observation − 0 vs. 2 device observations − 0 vs. 3 device observations	OR 4.6 (1.4–14.5) OR 14,9 (5.2–43.1) OR 42.4 (12.6–142.1)
Brasca F. 2016.	Customized	HF hospitalization (primary, though part of a composite endpoint)	OR 6.24 (4.90–7.95, *P* < 0.001)
Boehmer J. 2017.	HeartLogic	HF hospitalization (primary, though part of a composite endpoint)	Sensitivity 70%
Burri H. 2018.	TriageHF	HF hospitalization (secondary) - Low vs. high HFRS	RR 6.3 (3.9–10.2, *P* < 0.001)
Gardner R. 2018.	HeartLogic	HF hospitalization (primary, though part of a composite endpoint)	ERR 7.05 (4.69–10.61, *P* < 0.0001)
Okumura K. 2020.	TriageHF	HF hospitalization (primary) - Low vs. intermediate HFRS - Low vs. high HFRS	RR 2.18 (1.23–3.85) RR 5.78 (3.34–10.01)
Zile M. 2020.	TriageHF	HF hospitalization (primary) - Low vs. intermediate HFRS - Low vs. high HFRS	OR 2.8 (2.5–3.2, *P* < 0.001) OR 9.2 (8.1–10.3, *P* < 0.001)
Calo L. 2021.	HeartLogic	HF hospitalization (primary, though part of a composite endpoint)	HR 30.63 (13.04–71.95) *P* < 0.001
Baguda J. 2022.	HeartLogic	HF hospitalization (primary, though part of a composite endpoint)	Sensitivity 98%, specificity 90% PPV 29%, NPV 99.9%
D’Onofrio A. 2022.	HeartInsight	HF hospitalization (primary)	Sensitivity 65.5%, specificity 86.7%
Samut-Powell C. 2022.	TriageHF	HF hospitalization (secondary) - High vs. non-high HFRS	Sensitivity 62.5%, specificity 85.6%
Boriani G. 2023.	HeartLogic	HF hospitalization (primary) - In patients in sinus rhythm - In patients with atrial fibrillation	IRR 8.59 (1.67–55.31) IRR 2.70 (1.01–28.33)
Cardoso I. 2023.	TriageHF	HF hospitalization (primary) - Stepwise increase HFRS	OR 12.7 (3.2–51.5) *P* < 0.001
Singh J. 2024.	HeartLogic	HF hospitalization (primary, though part of a composite endpoint)	Sensitivity 74,5%
**QUESTION 3. UNPLANNED OUTPATIENT HF VISITS**			
Brasca F. 2016.	Customized	Unplanned visits (primary, though part of a composite endpoint)	OR 6.24 (4.90–7.95, *P* < 0.001)
Boehmer J. 2017.	HeartLogic	Unplanned visits (primary, though part of a composite endpoint)	Sensitivity 70%
Gardner R. 2018.	HeartLogic	Unplanned visits (primary, though part of a composite endpoint)	ERR 7.05 (4.69–10.61, *P* < 0.0001)
Baguda J. 2022.	HeartLogic	Unplanned visits (primary, though part of a composite endpoint)	Sensitivity 98%, specificity 90% PPV 29%, NPV 99.9%
Singh J. 2024.	HeartLogic	Unplanned visits (primary, though part of a composite endpoint)	Sensitivity 74,5%

Table 1: Available literature on the ability of CIED-based multisensor algorithms to discern patients at high risk for all-cause/cardiovascular mortality, heart failure hospitalization and unplanned outpatient heart failure visits. CV: cardiovascular. HR: hazard ratio. ERR: event rate ratio. IRR: incidence rate ratio. aHR: adjusted hazard ratio. HFRS: heart failure risk score. OR: odds ratio. PPV: positive predictive value. HF: heart failure. RR: relative risk. NPV: negative predictive value.

### Study Question 1: Multisensor, CIED-based HF Monitoring and Mortality

Thirteen out of the 30 articles included in this systematic review investigated all-cause and/or cardiovascular mortality as a primary or secondary endpoint, either independently or as part of a composite endpoint. Among these, eight studies evaluated whether CIED-based multisensor HF monitoring could identify patients at increased risk for mortality [21, 26–32]. The MultiSENSE study was the first to validate the HeartLogic algorithm’s effectiveness in this context [23, 26]. In a patient cohort of 900 HF-patients, this study showed that HF events (including all-cause mortality) were 10-times more frequent in patients with an ‘in alert’ status compared to patients ‘out of alert’ status. Various subsequent studies confirmed these findings [28, 30–32]. Similar results were observed for the TriageHF-algorithm [27, 29] and the HeartInsight-algorithm [21].

The remaining five studies, comprising two retrospective and three prospective investigations, examined the potential of multisensor algorithms in reducing all-cause and/or cardiovascular mortality [33–37]. In the randomized controlled IN-TIME trial, 716 patients with an Implantable Cardioverter Defibrillator (ICD) and Cardiac Resynchronization Therapy-Defibrillator (CRT-D) were randomly assigned to multisensor CIED-based monitoring or standard of care [33]. The primary outcome measure was a composite clinical score combining all-cause death, overnight hospital admission for HF, change in New York Heart Association (NYHA) class, and change in patient global self-assessment. At one year, the odds ratio (OR) for the primary endpoint was 0.63 (95% CI 0.43–0.90), with a significant better survival in the multisensor CIED-based monitoring group in a post hoc analysis. The Kaplan Meier estimated one-year all-cause mortality was 3.4% in the multisensor CIED-based monitoring group compared to 8.7% in the control group (*p* = 0.004). In contrast to the findings in the IN-TIME trial, the two other prospective trials with customized parameter designs, entitled MORE-CARE [34] and the REM-HF [35], could not demonstrate that multisensor CIED-based monitoring resulted in decreased mortality; the same was true for the two additional retrospective studies [36, 37]. The results of a meta-analysis of these five key studies are summarized in a forest plot (Fig. 3A), which shows a pooled IRR of 0.65 (95% CI: 0.40–1.06), reflecting no pooled reduction in mortality.

However, all five aforementioned trials used customized parameter designs. As of today, we must conclude that there are no prospective studies, let al.one randomized trials, evaluating mortality reduction driven by any of the commercially available algorithms. In this context it is notable that Baguda et al. recently published the first study to prospectively evaluate the HeartLogic algorithm and its impact on a composite endpoint of all-cause mortality and HF hospitalization, whereby each patient acted as his or her own historical control [38]. In a cohort of 392 patients a significant reduction in the composite endpoint was seen after activation of the HeartLogic algorithm (11% vs. 22%, *P* < 0.001). However, mortality was assessed exclusively during the post-activation period, potentially introducing survival bias.

### Study Question 2: Multisensor, CIED-based HF Monitoring and HF Hospitalizations

Twenty-three of the 30 examined studies incorporated HF hospitalizations as a primary or secondary outcome, either individually or within a composite endpoint. Most (sixteen) studies focused on the ability of CIED-based multisensor monitoring to identify patients at increased risk for a HF hospitalizations. Only seven studies explored the potential of CIED-based multisensor monitoring to reduce HF hospitalizations. Two of these seven studies were retrospective in nature [37, 39]. Among the remaining five prospective studies, two earlier RCTs employed customized parameter configurations [33, 34]. In these studies, Hindricks et al. and Boriani et al., were unable to demonstrate a significant reduction in HF hospitalizations when comparing CIED-based multisensor remote follow-up to standard care.

In contrast, the three more recent prospective studies used the HeartLogic [38, 40] and TriageHF algorithm [41], respectively. Treskes et al. reported that, in a cohort of 68 patients with historical controls, activation of the HeartLogic algorithm coincided with a significant decrease in hospitalizations for decompensated HF (7 episodes versus 27 episodes, *P* = 0.003) [40]. This was confirmed in a cohort of 392 patients with historical control, showing a significant reduction in the composite endpoint of HF hospitalizations and all-cause mortality after activation of the HeartLogic algorithm (11% vs. 22%, *P* < 0.001) [38]. Ahmed et al. showed that the use of the TriageHF Plus algorithm in a real-world cohort of 758 ICD and CRT patients resulted in a significant decrease in non-elective hospitalizations (all-cause, cardiovascular and/or heart failure related) after a median follow-up of 14 months (IRR 0.42 (95% CI 0.23–0.76, *P* = 0.004)), however the difference in HF hospitalizations was not significant (IRR 0.62 (95% CI 0.32–1.20)) [41]. The results of a meta-analysis of these seven studies are summarized in a forest plot (Fig. 3B), which shows a pooled IRR of 0.59 (95% CI: 0.39–0.88), reflecting statistically significant reduction in HF hospitalizations.

It is however important to note that there are no RCTs evaluating any of the three commercially available algorithms.

### Study Question 3: Multisensor, CIED-based HF Monitoring and Unplanned Outpatient HF Visits

The impact of CIED-based multisensor remote monitoring on unplanned outpatient HF visits has been less well studied. Only ten studies designated this aspect as an endpoint, either integrated within a composite primary endpoint framework or defined as a secondary endpoint. Notably, no studies were identified with unplanned outpatient HF visits as a distinct primary endpoint. Among the aforementioned ten studies, five investigated the efficacy of CIED-based multisensor remote monitoring in identifying patients at increased risk of unplanned outpatient HF visits, while the remaining five examined whether these algorithms could reduce the burden of such visits, of which three had a prospective design [34, 38, 42]. Two (earlier) RCTs investigated whether customized CIED-based algorithms could reduce the burden of unplanned outpatient HF visits. The EVOLVO trial, conducted in 200 patients with a CRT-D or ICD, revealed that remote monitoring, as opposed to standard management, significantly lowered the risk of emergency department visits or unplanned outpatient HF visits for HF exacerbations (IRR 0.52, 95% CI 0.37–0.73; *P* < 0.001) [42]. Similarly, the MORE CARE trial, involving 865 CRT-D patients randomized to either remote checks alternating with in-office follow-ups (remote arm) or in-office follow-ups alone, showed a substantially reduced risk of outpatient visits (both urgent and/or scheduled) associated with remote follow-up (HR 0.59, 95% CI 0.56–0.62; *P* < 0.001) [34]. More recently, Baguda et al. showed a significant reduction in unplanned outpatient HF visits after activation of the HeartLogic algorithm in a cohort of 392 patients with historical control (12% vs. 24%, *P* < 0.001) [38]. This was confirmed in 2 retrospective trials evaluating a customized parameter design and HeartLogic, respectively [37, 39]. The results of a meta-analysis of these five studies are summarized in a forest plot (Fig. 3C), which shows a pooled IRR of 0.59 (95% CI: 0.55–0.63), reflecting statistically significant reduction in unplanned outpatient HF visits.

No RCTs are yet available to confirm these results for any of the three commercially available algorithms.

## Discussion

This review is the first to provide an overview of the available evidence on the clinical impact of multiparametric CIED-based remote monitoring, including the commercially available algorithms HeartInsight, HeartLogic, and TriageHF. It highlights that while the older single-sensor monitoring approaches have had limited success in predicting and preventing HF events, the more advanced multisensor CIED-based algorithms effectively predict impeding HF events. Also, while older trials with customized multiparameter designs could not consistently show impact on the reduction of mortality, heart failure hospitalizations and/or outpatient visits, contemporary commercially available algorithms (mainly HeartLogic, and TriageHF) hold the potential to reduce their incidence, mainly HF hospitalization and unplanned urgent HF visits. However, several considerations should be made. First, as of today no RCTs are available to validate the benefit on clinical endpoints for these commercial algorithms and available observational data should therefore be interpreted with caution. RCTs are essential to solidify their role in structured management plans. Notably, the upcoming Phase 2 of the MANAGE-HF trial will assess HeartLogic’s impact on HF hospitalizations and mortality, potentially providing robust evidence for its role in HF management. Second, atrial fibrillation frequently coincides with HF and is known to affect disease severity and prognosis [43]. As atrial fibrillation might impact heart rate dependent variables of multisensory algorithms, it seems intuitive that its presence may impact and even drive alerts. In HeartLogic AT/AF burden is not part of the device algorithm (contrary to TriageHF and HeartInsight). However, Boriani et al. showed in a retrospective analysis of 568 patients with activated HeartLogic-algorithm more HeartLogic alerts during long-lasting episodes of atrial fibrillation, but a persisting ability to identify periods of increased risk of HF events [44]. Third, it is important to note that other types of (invasive) HF monitoring have also been developed. One example is hemodynamic monitoring using the CARDIOMEMS HF system (Abbott Laboratories, Abbott Park, Illionois, USA) or Cordella system (Endotronix, Woodridge, Illinois, USA), which is implanted in the pulmonary artery tree through a minimally invasive procedure. A recent meta-analysis found that hemodynamic-guided remote monitoring strategies significantly reduced the risk of HF hospitalizations compared to standard of care, with an IRR of 0.85 (95% CI 0.76–0.96), confirming the results of an earlier meta-analysis [19, 20]. However, unlike hemodynamic sensors, which require a specific implantation procedure, CIED-based algorithms are driven by devices already indicated for the patient, thereby avoiding any additional procedures and risks. Moreover, CIED-based algorithms are less relient on patient cooperation, unlike hemodynamic sensors, which require the patients to actively transmit the measurements. Also, the additional value of combining a hemodynamic sensor and multisensor CIED-based algorithm remains unclear and requires further investigation. Fourth, while CIED-based remote monitoring has advanced significantly over the past decade, it is the clinical response to alerts, not the alert itself, that drives improved outcomes. Currently, no standardized approach exists for managing these alerts. Most prospective trials applied predefined protocols involving actions like watchful waiting, phone contact, or additional clinic visits (Supplemental Table 4), but therapeutic decisions were often left to the treating physician, resulting in considerable variability. Key questions remain, such as whether diuretics should be initiated or adjusted even in asymptomatic patients, or whether priority should be given to optimizing disease-modifying therapy [45]. Few studies have addressed this. Phase 1 of the MANAGE-HF trial used a structured response algorithm for HeartLogic alerts and found this to be safe [46]. Similarly, the INTERVENE-HF study showed that a short course of intensified diuretics following TriageHF alerts restored thoracic impedance in most patients [47]. However, real-world implementation remains highly variable, which can significantly influence outcomes and limit reproducibility. It should thus be clear that the clinical success of these algorithms depends not only on technological performance, but also on integration into structured workflows that ensure timely and appropriate intervention. Lastly, this appropriate handling of alerts comes with considerable resource demands, as alerts require prompt triage, documentation, and follow-up, often by specialized HF nurses or technicians. In systems where reimbursement depends on documentation, this may impose a substantial administrative burden. The cost-effectiveness of algorithm-based heart failure management is therefore of particular interest, yet remains insufficiently studied. Recent guidance from the UK’s National Institute for Health and Care Excellence (NICE), however, suggests that HeartLogic and TriageHF may be both more effective and less costly than standard care, primarily due to reductions in HF hospitalizations [48]. These findings, based on observational data [40, 41], should be interpreted with caution, as their effectiveness and feasibility are highly dependent on the structure of the healthcare system and reimbursement models. Furthermore, the burden of false-positive alerts was only partially accounted for, potentially overestimating the overall benefit. For HeartInsight, the current lack of outcome data precludes any meaningful assessment of cost-effectiveness. However, looking ahead, the further integration of artificial intelligence into CIED-based monitoring systems might hold the potential to further enhance predictive accuracy by uncovering subtle, nonlinear patterns of decompensation. As training datasets grow and real-world implementation expands, artificial intelligence-driven algorithms may ultimately enable more precise and potentially less resource-intensive individualized remote heart failure management.

## Limitations

Several factors should be taken into account when interpreting the results of the current analysis. First, there are no RCTs validating the clinical impact of commercially available multisensor algorithms. As a result, the evidence relies heavily on observational studies, which are more prone to bias. Second, significant variability exists in the study designs, patient populations, and algorithms used, making it challenging to compare results and generalize findings. Third, the definitions of heart failure hospitalizations and outpatient visits were not always consistent across studies. Lastly, the clinical effectiveness of these algorithms depends on how alerts are managed, yet response protocols were not standardized and often left at the individual physician discretion, which limits the reproducibility and generalizability of the findings.

## Conclusion

This systematic review evaluates the clinical value of multisensor CIED-alert based heart failure management. Current built-in algorithms can reliably identify patients at increased risk for mortality, HF hospitalizations, and unplanned outpatient visits. However, the evidence supporting their ability to reduce these adverse outcomes remains limited. While our meta-analysis shows a reduction in HF hospitalizations and unplanned outpatient visits (particularly for HeartLogic), these findings currently lack prospective validation from RCTs. Moreover, the absence of a standardized approach to responding to device alerts remains a barrier for its generalizability. If validated in future RCTs, multisensor monitoring could become an essential tool in HF management, provided it is effectively integrated into structured clinical workflows.

## Key References

D’Onofrio A, Solimene F, Calò L, Calvi V, Viscusi M, Melissano D, et al. Combining home monitoring temporal trends from implanted defibrillators and baseline patient risk profile to predict heart failure hospitalizations: results from the SELENE HF study. Europace. 2022;24 [2]:234 − 44. **This paper was the landmark trial for the HeartInsight-algorithm.**

Cowie MR, Sarkar S, Koehler J, Whellan DJ, Crossley GH, Tang WH, et al. Development and validation of an integrated diagnostic algorithm derived from parameters monitored in implantable devices for identifying patients at risk for heart failure hospitalization in an ambulatory setting. Eur Heart J. 2013;34 [31]:2472-80. **This paper was the landmark trial for the TriageHF-algorithm.**

Boehmer JP, Hariharan R, Devecchi FG, Smith AL, Molon G, Capucci A, et al. A Multisensor Algorithm Predicts Heart Failure Events in Patients With Implanted Devices: Results From the MultiSENSE Study. JACC Heart Fail. 2017;5 [3]:216 − 25. **This paper was the landmark trial for the HeartLogic-algorithm.**

## Electronic Supplementary Material

Below is the link to the electronic supplementary material.

Supplementary Material 1

## Data Availability

No datasets were generated or analysed during the current study.
