# Supplementary material for: Impact of Multisensor CIED-based Heart Failure Monitoring on Mortality, Heart Failure Hospitalizations and Outpatient Visits: A Systematic Review
Source: Curr Heart Fail Rep. 2025 Jul 1;22(1):21. doi: 10.1007/s11897-025-00707-y (PMC12213901; doi:10.1007/s11897-025-00707-y)
Supplement: Supplementary file 1 — Supplementary Material 1 [file 11897_2025_707_MOESM1_ESM.docx]

**Supplementary materials**

**Impact of multisensor CIED-based heart failure monitoring on mortality, heart failure hospitalizations and outpatient visits: a systematic review.**

Bert Zwaenepoel, MD ^1, 2 *^; Annefleur Kluft, MSci ^2 *^; Michelle Feijen ^2^, MD, PhD; Jan W. Schoones ^3^, MA; Ward A. Heggermont, MD, PhD ^4^; Anastasia D. Egorova, MD, PhD ^2^ ^†^; Saskia L.M.A. Beeres, MD, PhD ^2^ ^†^

*1 Department of Cardiology, AZ Delta, Roeselare, Belgium*

*2 Department of Cardiology, Leiden University Medical Centre, Leiden, The Netherlands*

*3 Directorate of Research Policy, Leiden University Medical Center, Leiden, the Netherlands*

*4 Cardiovascular Research Centre Aalst, Department of Cardiology, OLV Clinic, Aalst, Belgium*

Inhoudsopgave

[Supplemental tables 3](#_Toc183436893)

[Supplemental Table 1: search strategy 3](#_Toc183436894)

[Supplemental Table 2: assessment of bias within studies 12](#_Toc183436895)

[Supplemental Table 3: general overview of all included articles 21](#_Toc183436896)

[Supplemental Table 4: interventions to device-based alerts 28](#_Toc183436897)

[Supplemental Table 5: PRISMA 2020 checklist 30](#_Toc183436898)

[Supplemental Table 6: PRIMSA 2020 checklist for abstracts 32](#_Toc183436899)

# Supplemental tables

## Supplemental Table 1: search strategy

| Databases searched | - PubMed - Embase - Web of Science - Cochrane Library - Emcare |
| --- | --- |
| Years of search | Published after January 1st of 2012 and before 3rd of July 2024 |
| Language | English language only |
| Search terms PubMed | ((("Heart Failure"[Mesh] OR "heart failure"[tw] OR "Cardio-Renal Syndrome"[tw] OR "Paroxysmal Dyspnea"[tw] OR "Cardiac Edema"[tw] OR "Cardiac Oedema"[tw] OR "Cardiac Failure"[tw] OR "Heart Decompensation"[tw] OR "Myocardial Failure"[tw]) AND ("Cardiac Resynchronization Therapy"[Mesh] OR "Defibrillators, Implantable"[Mesh] OR "implantable defibrillator"[tw] OR "implantable cardiac defibrillator"[tw] OR "implantable cardioverter-defibrillator"[tw] OR "cardiac implantable electronic device"[tw] OR "cardiac implanted electronic device"[tw] OR "implantable device"[tw] OR "cardiac device"[tw] OR "implantable cardioverter defibrillator"[tw] OR "implanted defibrillator"[tw] OR "cardiac resynchronisation therapy"[tw] OR "cardiac resynchronization therapy"[tw] OR "implantable defibrillators"[tw] OR "implantable cardiac defibrillators"[tw] OR "implantable cardioverter-defibrillators"[tw] OR "cardiac implantable electronic devices"[tw] OR "cardiac implanted electronic devices"[tw] OR "implantable devices"[tw] OR "cardiac devices"[tw] OR "implantable cardioverter defibrillators"[tw] OR "implanted defibrillators"[tw] OR "cardiac resynchronisation therap*"[tw] OR "cardiac resynchronization therap*"[tw] OR "implantable defibrillator*"[tw] OR "implantable cardiac defibrillator*"[tw] OR "implantable cardioverter-defibrillator*"[tw] OR "cardiac implantable electronic device*"[tw] OR "cardiac implanted electronic device*"[tw] OR "implantable device*"[tw] OR "cardiac device*"[tw] OR "implantable cardioverter defibrillator*"[tw] OR "implanted defibrillator*"[tw]) AND ("multisensor"[tw] OR "multisens*"[tw] OR "multi sensor"[tw] OR "multi sens*"[tw] OR "multiparametric"[tw] OR "multiparamet*"[tw] OR "multi parametric"[tw] OR "multi paramet*"[tw]) NOT (("Review"[pt] OR "Systematic Review"[pt] OR "Meta-Analysis"[pt] OR "Case Reports"[ptyp] OR "case report"[ti] OR "case rep"[all fields]) AND ("Clinical Study"[pt] OR "trial"[ti] OR "RCT"[ti])) NOT ("Animals"[mesh] NOT "Humans"[mesh])) OR (("Heart Failure"[majr] OR "heart failure"[tiab] OR "Cardio-Renal Syndrome"[tiab] OR "Paroxysmal Dyspnea"[tiab] OR "Cardiac Edema"[tiab] OR "Cardiac Oedema"[tiab] OR "Cardiac Failure"[tiab] OR "Heart Decompensation"[tiab] OR "Myocardial Failure"[tiab]) AND ("Cardiac Resynchronization Therapy"[majr] OR "Defibrillators, Implantable"[majr] OR "implantable defibrillator"[tiab] OR "implantable cardiac defibrillator"[tiab] OR "implantable cardioverter-defibrillator"[tiab] OR "cardiac implantable electronic device"[tiab] OR "cardiac implanted electronic device"[tiab] OR "implantable device"[tiab] OR "cardiac device"[tiab] OR "implantable cardioverter defibrillator"[tiab] OR "implanted defibrillator"[tiab] OR "cardiac resynchronisation therapy"[tiab] OR "cardiac resynchronization therapy"[tiab] OR "implantable defibrillators"[tiab] OR "implantable cardiac defibrillators"[tiab] OR "implantable cardioverter-defibrillators"[tiab] OR "cardiac implantable electronic devices"[tiab] OR "cardiac implanted electronic devices"[tiab] OR "implantable devices"[tiab] OR "cardiac devices"[tiab] OR "implantable cardioverter defibrillators"[tiab] OR "implanted defibrillators"[tiab] OR "cardiac resynchronisation therap*"[tiab] OR "cardiac resynchronization therap*"[tiab] OR "implantable defibrillator*"[tiab] OR "implantable cardiac defibrillator*"[tiab] OR "implantable cardioverter-defibrillator*"[tiab] OR "cardiac implantable electronic device*"[tiab] OR "cardiac implanted electronic device*"[tiab] OR "implantable device*"[tiab] OR "cardiac device*"[tiab] OR "implantable cardioverter defibrillator*"[tiab] OR "implanted defibrillator*"[tiab]) AND ("multisensor"[tiab] OR "multisens*"[tiab] OR "multi sensor"[tiab] OR "multi sens*"[tiab] OR "multiparametric"[tiab] OR "multiparamet*"[tiab] OR "multi parametric"[tiab] OR "multi paramet*"[tiab] OR "remote*"[tiab] OR "ambulator*"[tiab] OR "tele*"[tiab] OR "monitor*"[tiab] OR "Monitoring, Ambulatory"[Mesh] OR "Monitoring, Physiologic"[Mesh]) AND ("fluid retention"[tiab] OR "fluid status"[tiab] OR "fluid index"[tiab] OR "worsening"[tiab] OR "worse*"[tiab] OR "decompensation"[tiab] OR "congestion"[tiab] OR "congestive"[tiab] OR "Symptom Flare Up"[Mesh] OR "Disease Progression"[Mesh]) NOT (("Review"[pt] OR "Systematic Review"[pt] OR "Meta-Analysis"[pt] OR "Case Reports"[ptyp] OR "case report"[ti] OR "case rep"[all fields]) AND ("Clinical Study"[pt] OR "trial"[ti] OR "RCT"[ti])) NOT ("Animals"[mesh] NOT "Humans"[mesh])) OR (("Heart Failure"[majr] OR "heart failure"[ti] OR "Cardio-Renal Syndrome"[ti] OR "Paroxysmal Dyspnea"[ti] OR "Cardiac Edema"[ti] OR "Cardiac Oedema"[ti] OR "Cardiac Failure"[ti] OR "Heart Decompensation"[ti] OR "Myocardial Failure"[ti]) AND ("Cardiac Resynchronization Therapy"[majr] OR "Defibrillators, Implantable"[majr] OR "implantable defibrillator"[ti] OR "implantable cardiac defibrillator"[ti] OR "implantable cardioverter-defibrillator"[ti] OR "cardiac implantable electronic device"[ti] OR "cardiac implanted electronic device"[ti] OR "implantable device"[ti] OR "cardiac device"[ti] OR "implantable cardioverter defibrillator"[ti] OR "implanted defibrillator"[ti] OR "cardiac resynchronisation therapy"[ti] OR "cardiac resynchronization therapy"[ti] OR "implantable defibrillators"[ti] OR "implantable cardiac defibrillators"[ti] OR "implantable cardioverter-defibrillators"[ti] OR "cardiac implantable electronic devices"[ti] OR "cardiac implanted electronic devices"[ti] OR "implantable devices"[ti] OR "cardiac devices"[ti] OR "implantable cardioverter defibrillators"[ti] OR "implanted defibrillators"[ti] OR "cardiac resynchronisation therap*"[ti] OR "cardiac resynchronization therap*"[ti] OR "implantable defibrillator*"[ti] OR "implantable cardiac defibrillator*"[ti] OR "implantable cardioverter-defibrillator*"[ti] OR "cardiac implantable electronic device*"[ti] OR "cardiac implanted electronic device*"[ti] OR "implantable device*"[ti] OR "cardiac device*"[ti] OR "implantable cardioverter defibrillator*"[ti] OR "implanted defibrillator*"[ti]) AND ("multisensor"[tiab] OR "multisens*"[tiab] OR "multi sensor"[tiab] OR "multi sens*"[tiab] OR "multiparametric"[tiab] OR "multiparamet*"[tiab] OR "multi parametric"[tiab] OR "multi paramet*"[tiab] OR "remote monitoring"[tiab] OR "ambulatory monitoring"[tiab] OR "telemonitoring"[tiab] OR "monitoring"[tiab] OR "remote monitor*"[tiab] OR "ambulatory monitor*"[tiab] OR "telemonitor*"[tiab] OR (("monitor"[ti] OR "monitor*"[ti] OR "Monitoring, Ambulatory"[mesh] OR "Monitoring, Physiologic"[majr]) AND ("Algorithms"[Mesh] OR "Algorithms"[tiab] OR "Algorithm"[tiab] OR "Algorithm*"[tiab] OR "Artificial Intelligence"[tiab] OR "Machine Learning"[tiab] OR "Deep Learning"[tiab]))) NOT (("Review"[pt] OR "Systematic Review"[pt] OR "Meta-Analysis"[pt] OR "Case Reports"[ptyp] OR "case report"[ti] OR "case rep"[all fields]) AND ("Clinical Study"[pt] OR "trial"[ti] OR "RCT"[ti])) NOT ("Animals"[mesh] NOT "Humans"[mesh])) OR (("HeartLogic"[tw] OR "Heart Logic"[tw] OR "Triage HF"[tw] OR "TriageHF"[tw] OR "HeartInsight"[tw]) NOT (("Review"[pt] OR "Systematic Review"[pt] OR "Meta-Analysis"[pt] OR "Case Reports"[ptyp] OR "case report"[ti] OR "case rep"[all fields]) AND ("Clinical Study"[pt] OR "trial"[ti] OR "RCT"[ti])) NOT ("Animals"[mesh] NOT "Humans"[mesh]))) AND ("2012/01/01"[PDAT] : "3000/12/31"[PDAT]) |
| Search terms Embase | (((exp *"Heart Failure"/ OR "heart failure".ti,ab OR "Cardio-Renal Syndrome".ti,ab OR "Paroxysmal Dyspnea".ti,ab OR "Cardiac Edema".ti,ab OR "Cardiac Oedema".ti,ab OR "Cardiac Failure".ti,ab OR "Heart Decompensation".ti,ab OR "Myocardial Failure".ti,ab) AND (exp *"Cardiac Resynchronization Therapy"/ OR exp *"Implantable Cardioverter Defibrillators"/ OR "implantable defibrillator".ti,ab OR "implantable cardiac defibrillator".ti,ab OR "implantable cardioverter-defibrillator".ti,ab OR "cardiac implantable electronic device".ti,ab OR "cardiac implanted electronic device".ti,ab OR "implantable device".ti,ab OR "cardiac device".ti,ab OR "implantable cardioverter defibrillator".ti,ab OR "implanted defibrillator".ti,ab OR "cardiac resynchronisation therapy".ti,ab OR "cardiac resynchronization therapy".ti,ab OR "implantable defibrillators".ti,ab OR "implantable cardiac defibrillators".ti,ab OR "implantable cardioverter-defibrillators".ti,ab OR "cardiac implantable electronic devices".ti,ab OR "cardiac implanted electronic devices".ti,ab OR "implantable devices".ti,ab OR "cardiac devices".ti,ab OR "implantable cardioverter defibrillators".ti,ab OR "implanted defibrillators".ti,ab OR "cardiac resynchronisation therap*".ti,ab OR "cardiac resynchronization therap*".ti,ab OR "implantable defibrillator*".ti,ab OR "implantable cardiac defibrillator*".ti,ab OR "implantable cardioverter-defibrillator*".ti,ab OR "cardiac implantable electronic device*".ti,ab OR "cardiac implanted electronic device*".ti,ab OR "implantable device*".ti,ab OR "cardiac device*".ti,ab OR "implantable cardioverter defibrillator*".ti,ab OR "implanted defibrillator*".ti,ab) AND ("multisensor".ti,ab OR "multisens*".ti,ab OR "multi sensor".ti,ab OR "multi sens*".ti,ab OR "multiparametric".ti,ab OR "multiparamet*".ti,ab OR "multi parametric".ti,ab OR "multi paramet*".ti,ab)) OR ((exp *"Heart Failure"/ OR "heart failure".ti,ab OR "Cardio-Renal Syndrome".ti,ab OR "Paroxysmal Dyspnea".ti,ab OR "Cardiac Edema".ti,ab OR "Cardiac Oedema".ti,ab OR "Cardiac Failure".ti,ab OR "Heart Decompensation".ti,ab OR "Myocardial Failure".ti,ab) AND (exp *"Cardiac Resynchronization Therapy"/ OR exp *"Implantable Cardioverter Defibrillators"/ OR "implantable defibrillator".ti,ab OR "implantable cardiac defibrillator".ti,ab OR "implantable cardioverter-defibrillator".ti,ab OR "cardiac implantable electronic device".ti,ab OR "cardiac implanted electronic device".ti,ab OR "implantable device".ti,ab OR "cardiac device".ti,ab OR "implantable cardioverter defibrillator".ti,ab OR "implanted defibrillator".ti,ab OR "cardiac resynchronisation therapy".ti,ab OR "cardiac resynchronization therapy".ti,ab OR "implantable defibrillators".ti,ab OR "implantable cardiac defibrillators".ti,ab OR "implantable cardioverter-defibrillators".ti,ab OR "cardiac implantable electronic devices".ti,ab OR "cardiac implanted electronic devices".ti,ab OR "implantable devices".ti,ab OR "cardiac devices".ti,ab OR "implantable cardioverter defibrillators".ti,ab OR "implanted defibrillators".ti,ab OR "cardiac resynchronisation therap*".ti,ab OR "cardiac resynchronization therap*".ti,ab OR "implantable defibrillator*".ti,ab OR "implantable cardiac defibrillator*".ti,ab OR "implantable cardioverter-defibrillator*".ti,ab OR "cardiac implantable electronic device*".ti,ab OR "cardiac implanted electronic device*".ti,ab OR "implantable device*".ti,ab OR "cardiac device*".ti,ab OR "implantable cardioverter defibrillator*".ti,ab OR "implanted defibrillator*".ti,ab) AND ("multisensor".ti,ab OR "multisens*".ti,ab OR "multi sensor".ti,ab OR "multi sens*".ti,ab OR "multiparametric".ti,ab OR "multiparamet*".ti,ab OR "multi parametric".ti,ab OR "multi paramet*".ti,ab OR "remote*".ti,ab OR "ambulator*".ti,ab OR "tele*".ti,ab OR "monitor*".ti,ab OR exp *"Ambulatory Monitoring"/ OR exp *"Physiologic Monitoring"/) AND ("fluid retention".ti,ab OR exp *"Fluid Retention"/ OR "fluid status".ti,ab OR "fluid index".ti,ab OR "worsening".ti,ab OR "worse*".ti,ab OR "decompensation".ti,ab OR "congestion".ti,ab OR "congestive".ti,ab OR "Flare Up".ti,ab OR "Disease Exacerbation"/)) OR ((exp *"Heart Failure"/ OR "heart failure".ti OR "Cardio-Renal Syndrome".ti OR "Paroxysmal Dyspnea".ti OR "Cardiac Edema".ti OR "Cardiac Oedema".ti OR "Cardiac Failure".ti OR "Heart Decompensation".ti OR "Myocardial Failure".ti) AND (exp *"Cardiac Resynchronization Therapy"/ OR exp *"Implantable Cardioverter Defibrillators"/ OR "implantable defibrillator".ti OR "implantable cardiac defibrillator".ti OR "implantable cardioverter-defibrillator".ti OR "cardiac implantable electronic device".ti OR "cardiac implanted electronic device".ti OR "implantable device".ti OR "cardiac device".ti OR "implantable cardioverter defibrillator".ti OR "implanted defibrillator".ti OR "cardiac resynchronisation therapy".ti OR "cardiac resynchronization therapy".ti OR "implantable defibrillators".ti OR "implantable cardiac defibrillators".ti OR "implantable cardioverter-defibrillators".ti OR "cardiac implantable electronic devices".ti OR "cardiac implanted electronic devices".ti OR "implantable devices".ti OR "cardiac devices".ti OR "implantable cardioverter defibrillators".ti OR "implanted defibrillators".ti OR "cardiac resynchronisation therap*".ti OR "cardiac resynchronization therap*".ti OR "implantable defibrillator*".ti OR "implantable cardiac defibrillator*".ti OR "implantable cardioverter-defibrillator*".ti OR "cardiac implantable electronic device*".ti OR "cardiac implanted electronic device*".ti OR "implantable device*".ti OR "cardiac device*".ti OR "implantable cardioverter defibrillator*".ti OR "implanted defibrillator*".ti) AND ("multisensor".ti,ab OR "multisens*".ti,ab OR "multi sensor".ti,ab OR "multi sens*".ti,ab OR "multiparametric".ti,ab OR "multiparamet*".ti,ab OR "multi parametric".ti,ab OR "multi paramet*".ti,ab OR "remote monitoring".ti,ab OR "ambulatory monitoring".ti,ab OR "telemonitoring".ti,ab OR "monitoring".ti,ab OR "remote monitor*".ti,ab OR "ambulatory monitor*".ti,ab OR "telemonitor*".ti,ab OR (("monitor".ti OR "monitor*".ti OR exp *"Ambulatory Monitoring"/ OR exp *"Physiologic Monitoring"/) AND (exp *"Algorithm"/ OR exp *"Machine Learning"/ OR "Algorithms".ti,ab OR "Algorithm".ti,ab OR "Algorithm*".ti,ab OR "Artificial Intelligence".ti,ab OR "Machine Learning".ti,ab OR "Deep Learning".ti,ab)))) OR (("HeartLogic".ti,ab OR "Heart Logic".ti,ab OR "Triage HF".ti,ab OR "TriageHF".ti,ab OR "HeartInsight".ti,ab))) NOT ((exp "Review"/ OR exp "Systematic Review"/ OR exp "Meta Analysis"/ OR "Case Report"/ OR "case report".ti OR (case AND (report OR reports)).jw.) NOT ("Clinical Study"/ OR "trial".ti OR "RCT".ti)) NOT (exp "Animals"/ NOT exp "Humans"/) AND 2012:2025.(sa_year) |
| Search terms Web of Science | (((TI=("Heart Failure" OR "heart failure" OR "Cardio-Renal Syndrome" OR "Paroxysmal Dyspnea" OR "Cardiac Edema" OR "Cardiac Oedema" OR "Cardiac Failure" OR "Heart Decompensation" OR "Myocardial Failure") OR AK=("Heart Failure" OR "heart failure" OR "Cardio-Renal Syndrome" OR "Paroxysmal Dyspnea" OR "Cardiac Edema" OR "Cardiac Oedema" OR "Cardiac Failure" OR "Heart Decompensation" OR "Myocardial Failure") OR AB=("Heart Failure" OR "heart failure" OR "Cardio-Renal Syndrome" OR "Paroxysmal Dyspnea" OR "Cardiac Edema" OR "Cardiac Oedema" OR "Cardiac Failure" OR "Heart Decompensation" OR "Myocardial Failure")) AND (TI=("Cardiac Resynchronization Therapy" OR "Implantable Cardioverter Defibrillators" OR "implantable defibrillator" OR "implantable cardiac defibrillator" OR "implantable cardioverter-defibrillator" OR "cardiac implantable electronic device" OR "cardiac implanted electronic device" OR "implantable device" OR "cardiac device" OR "implantable cardioverter defibrillator" OR "implanted defibrillator" OR "cardiac resynchronisation therapy" OR "cardiac resynchronization therapy" OR "implantable defibrillators" OR "implantable cardiac defibrillators" OR "implantable cardioverter-defibrillators" OR "cardiac implantable electronic devices" OR "cardiac implanted electronic devices" OR "implantable devices" OR "cardiac devices" OR "implantable cardioverter defibrillators" OR "implanted defibrillators" OR "cardiac resynchronisation therap*" OR "cardiac resynchronization therap*" OR "implantable defibrillator*" OR "implantable cardiac defibrillator*" OR "implantable cardioverter-defibrillator*" OR "cardiac implantable electronic device*" OR "cardiac implanted electronic device*" OR "implantable device*" OR "cardiac device*" OR "implantable cardioverter defibrillator*" OR "implanted defibrillator*") OR AK=("Cardiac Resynchronization Therapy" OR "Implantable Cardioverter Defibrillators" OR "implantable defibrillator" OR "implantable cardiac defibrillator" OR "implantable cardioverter-defibrillator" OR "cardiac implantable electronic device" OR "cardiac implanted electronic device" OR "implantable device" OR "cardiac device" OR "implantable cardioverter defibrillator" OR "implanted defibrillator" OR "cardiac resynchronisation therapy" OR "cardiac resynchronization therapy" OR "implantable defibrillators" OR "implantable cardiac defibrillators" OR "implantable cardioverter-defibrillators" OR "cardiac implantable electronic devices" OR "cardiac implanted electronic devices" OR "implantable devices" OR "cardiac devices" OR "implantable cardioverter defibrillators" OR "implanted defibrillators" OR "cardiac resynchronisation therap*" OR "cardiac resynchronization therap*" OR "implantable defibrillator*" OR "implantable cardiac defibrillator*" OR "implantable cardioverter-defibrillator*" OR "cardiac implantable electronic device*" OR "cardiac implanted electronic device*" OR "implantable device*" OR "cardiac device*" OR "implantable cardioverter defibrillator*" OR "implanted defibrillator*") OR AB=("Cardiac Resynchronization Therapy" OR "Implantable Cardioverter Defibrillators" OR "implantable defibrillator" OR "implantable cardiac defibrillator" OR "implantable cardioverter-defibrillator" OR "cardiac implantable electronic device" OR "cardiac implanted electronic device" OR "implantable device" OR "cardiac device" OR "implantable cardioverter defibrillator" OR "implanted defibrillator" OR "cardiac resynchronisation therapy" OR "cardiac resynchronization therapy" OR "implantable defibrillators" OR "implantable cardiac defibrillators" OR "implantable cardioverter-defibrillators" OR "cardiac implantable electronic devices" OR "cardiac implanted electronic devices" OR "implantable devices" OR "cardiac devices" OR "implantable cardioverter defibrillators" OR "implanted defibrillators" OR "cardiac resynchronisation therap*" OR "cardiac resynchronization therap*" OR "implantable defibrillator*" OR "implantable cardiac defibrillator*" OR "implantable cardioverter-defibrillator*" OR "cardiac implantable electronic device*" OR "cardiac implanted electronic device*" OR "implantable device*" OR "cardiac device*" OR "implantable cardioverter defibrillator*" OR "implanted defibrillator*")) AND TS=("multisensor" OR "multisens*" OR "multi sensor" OR "multi sens*" OR "multiparametric" OR "multiparamet*" OR "multi parametric" OR "multi paramet*")) OR ((TI=("Heart Failure" OR "heart failure" OR "Cardio-Renal Syndrome" OR "Paroxysmal Dyspnea" OR "Cardiac Edema" OR "Cardiac Oedema" OR "Cardiac Failure" OR "Heart Decompensation" OR "Myocardial Failure") OR AK=("Heart Failure" OR "heart failure" OR "Cardio-Renal Syndrome" OR "Paroxysmal Dyspnea" OR "Cardiac Edema" OR "Cardiac Oedema" OR "Cardiac Failure" OR "Heart Decompensation" OR "Myocardial Failure") OR AB=("Heart Failure" OR "heart failure" OR "Cardio-Renal Syndrome" OR "Paroxysmal Dyspnea" OR "Cardiac Edema" OR "Cardiac Oedema" OR "Cardiac Failure" OR "Heart Decompensation" OR "Myocardial Failure")) AND (TI=("Cardiac Resynchronization Therapy" OR "Implantable Cardioverter Defibrillators" OR "implantable defibrillator" OR "implantable cardiac defibrillator" OR "implantable cardioverter-defibrillator" OR "cardiac implantable electronic device" OR "cardiac implanted electronic device" OR "implantable device" OR "cardiac device" OR "implantable cardioverter defibrillator" OR "implanted defibrillator" OR "cardiac resynchronisation therapy" OR "cardiac resynchronization therapy" OR "implantable defibrillators" OR "implantable cardiac defibrillators" OR "implantable cardioverter-defibrillators" OR "cardiac implantable electronic devices" OR "cardiac implanted electronic devices" OR "implantable devices" OR "cardiac devices" OR "implantable cardioverter defibrillators" OR "implanted defibrillators" OR "cardiac resynchronisation therap*" OR "cardiac resynchronization therap*" OR "implantable defibrillator*" OR "implantable cardiac defibrillator*" OR "implantable cardioverter-defibrillator*" OR "cardiac implantable electronic device*" OR "cardiac implanted electronic device*" OR "implantable device*" OR "cardiac device*" OR "implantable cardioverter defibrillator*" OR "implanted defibrillator*") OR AK=("Cardiac Resynchronization Therapy" OR "Implantable Cardioverter Defibrillators" OR "implantable defibrillator" OR "implantable cardiac defibrillator" OR "implantable cardioverter-defibrillator" OR "cardiac implantable electronic device" OR "cardiac implanted electronic device" OR "implantable device" OR "cardiac device" OR "implantable cardioverter defibrillator" OR "implanted defibrillator" OR "cardiac resynchronisation therapy" OR "cardiac resynchronization therapy" OR "implantable defibrillators" OR "implantable cardiac defibrillators" OR "implantable cardioverter-defibrillators" OR "cardiac implantable electronic devices" OR "cardiac implanted electronic devices" OR "implantable devices" OR "cardiac devices" OR "implantable cardioverter defibrillators" OR "implanted defibrillators" OR "cardiac resynchronisation therap*" OR "cardiac resynchronization therap*" OR "implantable defibrillator*" OR "implantable cardiac defibrillator*" OR "implantable cardioverter-defibrillator*" OR "cardiac implantable electronic device*" OR "cardiac implanted electronic device*" OR "implantable device*" OR "cardiac device*" OR "implantable cardioverter defibrillator*" OR "implanted defibrillator*") OR AB=("Cardiac Resynchronization Therapy" OR "Implantable Cardioverter Defibrillators" OR "implantable defibrillator" OR "implantable cardiac defibrillator" OR "implantable cardioverter-defibrillator" OR "cardiac implantable electronic device" OR "cardiac implanted electronic device" OR "implantable device" OR "cardiac device" OR "implantable cardioverter defibrillator" OR "implanted defibrillator" OR "cardiac resynchronisation therapy" OR "cardiac resynchronization therapy" OR "implantable defibrillators" OR "implantable cardiac defibrillators" OR "implantable cardioverter-defibrillators" OR "cardiac implantable electronic devices" OR "cardiac implanted electronic devices" OR "implantable devices" OR "cardiac devices" OR "implantable cardioverter defibrillators" OR "implanted defibrillators" OR "cardiac resynchronisation therap*" OR "cardiac resynchronization therap*" OR "implantable defibrillator*" OR "implantable cardiac defibrillator*" OR "implantable cardioverter-defibrillator*" OR "cardiac implantable electronic device*" OR "cardiac implanted electronic device*" OR "implantable device*" OR "cardiac device*" OR "implantable cardioverter defibrillator*" OR "implanted defibrillator*")) AND TS=("multisensor" OR "multisens*" OR "multi sensor" OR "multi sens*" OR "multiparametric" OR "multiparamet*" OR "multi parametric" OR "multi paramet*" OR "remote*" OR "ambulator*" OR "tele*" OR "monitor*" OR "Ambulatory Monitoring" OR "Physiologic Monitoring") AND TS=("fluid retention" OR "Fluid Retention" OR "fluid status" OR "fluid index" OR "worsening" OR "worse*" OR "decompensation" OR "congestion" OR "congestive" OR "Flare Up" OR "Disease Exacerbation")) OR (TI=("Heart Failure" OR "heart failure" OR "Cardio-Renal Syndrome" OR "Paroxysmal Dyspnea" OR "Cardiac Edema" OR "Cardiac Oedema" OR "Cardiac Failure" OR "Heart Decompensation" OR "Myocardial Failure") AND TI=("Cardiac Resynchronization Therapy" OR "Implantable Cardioverter Defibrillators" OR "implantable defibrillator" OR "implantable cardiac defibrillator" OR "implantable cardioverter-defibrillator" OR "cardiac implantable electronic device" OR "cardiac implanted electronic device" OR "implantable device" OR "cardiac device" OR "implantable cardioverter defibrillator" OR "implanted defibrillator" OR "cardiac resynchronisation therapy" OR "cardiac resynchronization therapy" OR "implantable defibrillators" OR "implantable cardiac defibrillators" OR "implantable cardioverter-defibrillators" OR "cardiac implantable electronic devices" OR "cardiac implanted electronic devices" OR "implantable devices" OR "cardiac devices" OR "implantable cardioverter defibrillators" OR "implanted defibrillators" OR "cardiac resynchronisation therap*" OR "cardiac resynchronization therap*" OR "implantable defibrillator*" OR "implantable cardiac defibrillator*" OR "implantable cardioverter-defibrillator*" OR "cardiac implantable electronic device*" OR "cardiac implanted electronic device*" OR "implantable device*" OR "cardiac device*" OR "implantable cardioverter defibrillator*" OR "implanted defibrillator*") AND TS=("multisensor" OR "multisens*" OR "multi sensor" OR "multi sens*" OR "multiparametric" OR "multiparamet*" OR "multi parametric" OR "multi paramet*" OR "remote monitoring" OR "ambulatory monitoring" OR "telemonitoring" OR "monitoring" OR "remote monitor*" OR "ambulatory monitor*" OR "telemonitor*" OR (("monitor" OR "monitor*" OR "Ambulatory Monitoring" OR "Physiologic Monitoring") AND ("Algorithm" OR "Machine Learning" OR "Algorithms" OR "Algorithm" OR "Algorithm*" OR "Artificial Intelligence" OR "Machine Learning" OR "Deep Learning")))) OR (TS=("HeartLogic" OR "Heart Logic" OR "Triage HF" OR "TriageHF" OR "HeartInsight"))) NOT (TI=("Review" OR "Meta Analysis" OR "Case Report") NOT TI=("veterinary" OR "rabbit" OR "rabbits" OR "animal" OR "animals" OR "mouse" OR "mice" OR "rodent" OR "rodents" OR "rat" OR "rats" OR "pig" OR "pigs" OR "porcine" OR "horse" OR "horses" OR "equine" OR "cow" OR "cows" OR "bovine" OR "goat" OR "goats" OR "sheep" OR "ovine" OR "canine" OR "dog" OR "dogs" OR "feline" OR "cat" OR "cats") OR AK=("veterinary" OR "rabbit" OR "rabbits" OR "animal" OR "animals" OR "mouse" OR "mice" OR "rodent" OR "rodents" OR "rat" OR "rats" OR "pig" OR "pigs" OR "porcine" OR "horse" OR "horses" OR "equine" OR "cow" OR "cows" OR "bovine" OR "goat" OR "goats" OR "sheep" OR "ovine" OR "canine" OR "dog" OR "dogs" OR "feline" OR "cat" OR "cats")) AND PY=(2012 OR 2013 OR 2014 OR 2015 OR 2016 OR 2017 OR 2018 OR 2019 OR 2020 OR 2021 OR 2022 OR 2023 OR 2024 OR 2025) |
| Search terms Cochrane | ((("Heart Failure" OR "heart failure" OR "Cardio-Renal Syndrome" OR "Paroxysmal Dyspnea" OR "Cardiac Edema" OR "Cardiac Oedema" OR "Cardiac Failure" OR "Heart Decompensation" OR "Myocardial Failure"):ti,ab,kw AND ("Cardiac Resynchronization Therapy" OR "Implantable Cardioverter Defibrillators" OR "implantable defibrillator" OR "implantable cardiac defibrillator" OR "implantable cardioverter-defibrillator" OR "cardiac implantable electronic device" OR "cardiac implanted electronic device" OR "implantable device" OR "cardiac device" OR "implantable cardioverter defibrillator" OR "implanted defibrillator" OR "cardiac resynchronisation therapy" OR "cardiac resynchronization therapy" OR "implantable defibrillators" OR "implantable cardiac defibrillators" OR "implantable cardioverter-defibrillators" OR "cardiac implantable electronic devices" OR "cardiac implanted electronic devices" OR "implantable devices" OR "cardiac devices" OR "implantable cardioverter defibrillators" OR "implanted defibrillators" OR "cardiac resynchronisation therap*" OR "cardiac resynchronization therap*" OR "implantable defibrillator*" OR "implantable cardiac defibrillator*" OR "implantable cardioverter-defibrillator*" OR "cardiac implantable electronic device*" OR "cardiac implanted electronic device*" OR "implantable device*" OR "cardiac device*" OR "implantable cardioverter defibrillator*" OR "implanted defibrillator*"):ti,ab,kw AND ("multisensor" OR "multisens*" OR "multi sensor" OR "multi sens*" OR "multiparametric" OR "multiparamet*" OR "multi parametric" OR "multi paramet*"):ti,ab,kw) OR (("Heart Failure" OR "heart failure" OR "Cardio-Renal Syndrome" OR "Paroxysmal Dyspnea" OR "Cardiac Edema" OR "Cardiac Oedema" OR "Cardiac Failure" OR "Heart Decompensation" OR "Myocardial Failure"):ti,ab,kw AND ("Cardiac Resynchronization Therapy" OR "Implantable Cardioverter Defibrillators" OR "implantable defibrillator" OR "implantable cardiac defibrillator" OR "implantable cardioverter-defibrillator" OR "cardiac implantable electronic device" OR "cardiac implanted electronic device" OR "implantable device" OR "cardiac device" OR "implantable cardioverter defibrillator" OR "implanted defibrillator" OR "cardiac resynchronisation therapy" OR "cardiac resynchronization therapy" OR "implantable defibrillators" OR "implantable cardiac defibrillators" OR "implantable cardioverter-defibrillators" OR "cardiac implantable electronic devices" OR "cardiac implanted electronic devices" OR "implantable devices" OR "cardiac devices" OR "implantable cardioverter defibrillators" OR "implanted defibrillators" OR "cardiac resynchronisation therap*" OR "cardiac resynchronization therap*" OR "implantable defibrillator*" OR "implantable cardiac defibrillator*" OR "implantable cardioverter-defibrillator*" OR "cardiac implantable electronic device*" OR "cardiac implanted electronic device*" OR "implantable device*" OR "cardiac device*" OR "implantable cardioverter defibrillator*" OR "implanted defibrillator*"):ti,ab,kw AND ("multisensor" OR "multisens*" OR "multi sensor" OR "multi sens*" OR "multiparametric" OR "multiparamet*" OR "multi parametric" OR "multi paramet*" OR "remote*" OR "ambulator*" OR "tele*" OR "monitor*" OR "Ambulatory Monitoring" OR "Physiologic Monitoring"):ti,ab,kw AND ("fluid retention" OR "Fluid Retention" OR "fluid status" OR "fluid index" OR "worsening" OR "worse*" OR "decompensation" OR "congestion" OR "congestive" OR "Flare Up" OR "Disease Exacerbation"):ti,ab,kw) OR (("Heart Failure" OR "heart failure" OR "Cardio-Renal Syndrome" OR "Paroxysmal Dyspnea" OR "Cardiac Edema" OR "Cardiac Oedema" OR "Cardiac Failure" OR "Heart Decompensation" OR "Myocardial Failure"):ti AND ("Cardiac Resynchronization Therapy" OR "Implantable Cardioverter Defibrillators" OR "implantable defibrillator" OR "implantable cardiac defibrillator" OR "implantable cardioverter-defibrillator" OR "cardiac implantable electronic device" OR "cardiac implanted electronic device" OR "implantable device" OR "cardiac device" OR "implantable cardioverter defibrillator" OR "implanted defibrillator" OR "cardiac resynchronisation therapy" OR "cardiac resynchronization therapy" OR "implantable defibrillators" OR "implantable cardiac defibrillators" OR "implantable cardioverter-defibrillators" OR "cardiac implantable electronic devices" OR "cardiac implanted electronic devices" OR "implantable devices" OR "cardiac devices" OR "implantable cardioverter defibrillators" OR "implanted defibrillators" OR "cardiac resynchronisation therap*" OR "cardiac resynchronization therap*" OR "implantable defibrillator*" OR "implantable cardiac defibrillator*" OR "implantable cardioverter-defibrillator*" OR "cardiac implantable electronic device*" OR "cardiac implanted electronic device*" OR "implantable device*" OR "cardiac device*" OR "implantable cardioverter defibrillator*" OR "implanted defibrillator*"):ti AND ("multisensor" OR "multisens*" OR "multi sensor" OR "multi sens*" OR "multiparametric" OR "multiparamet*" OR "multi parametric" OR "multi paramet*" OR "remote monitoring" OR "ambulatory monitoring" OR "telemonitoring" OR "monitoring" OR "remote monitor*" OR "ambulatory monitor*" OR "telemonitor*" OR (("monitor" OR "monitor*" OR "Ambulatory Monitoring" OR "Physiologic Monitoring") AND ("Algorithm" OR "Machine Learning" OR "Algorithms" OR "Algorithm" OR "Algorithm*" OR "Artificial Intelligence" OR "Machine Learning" OR "Deep Learning"))):ti,ab,kw) OR (("HeartLogic" OR "Heart Logic" OR "Triage HF" OR "TriageHF" OR "HeartInsight"):ti,ab,kw)) |
| Search terms Emcare | (((exp *"Heart Failure"/ OR "heart failure".ti,ab OR "Cardio-Renal Syndrome".ti,ab OR "Paroxysmal Dyspnea".ti,ab OR "Cardiac Edema".ti,ab OR "Cardiac Oedema".ti,ab OR "Cardiac Failure".ti,ab OR "Heart Decompensation".ti,ab OR "Myocardial Failure".ti,ab) AND (exp *"Cardiac Resynchronization Therapy"/ OR exp *"Implantable Cardioverter Defibrillators"/ OR "implantable defibrillator".ti,ab OR "implantable cardiac defibrillator".ti,ab OR "implantable cardioverter-defibrillator".ti,ab OR "cardiac implantable electronic device".ti,ab OR "cardiac implanted electronic device".ti,ab OR "implantable device".ti,ab OR "cardiac device".ti,ab OR "implantable cardioverter defibrillator".ti,ab OR "implanted defibrillator".ti,ab OR "cardiac resynchronisation therapy".ti,ab OR "cardiac resynchronization therapy".ti,ab OR "implantable defibrillators".ti,ab OR "implantable cardiac defibrillators".ti,ab OR "implantable cardioverter-defibrillators".ti,ab OR "cardiac implantable electronic devices".ti,ab OR "cardiac implanted electronic devices".ti,ab OR "implantable devices".ti,ab OR "cardiac devices".ti,ab OR "implantable cardioverter defibrillators".ti,ab OR "implanted defibrillators".ti,ab OR "cardiac resynchronisation therap*".ti,ab OR "cardiac resynchronization therap*".ti,ab OR "implantable defibrillator*".ti,ab OR "implantable cardiac defibrillator*".ti,ab OR "implantable cardioverter-defibrillator*".ti,ab OR "cardiac implantable electronic device*".ti,ab OR "cardiac implanted electronic device*".ti,ab OR "implantable device*".ti,ab OR "cardiac device*".ti,ab OR "implantable cardioverter defibrillator*".ti,ab OR "implanted defibrillator*".ti,ab) AND ("multisensor".ti,ab OR "multisens*".ti,ab OR "multi sensor".ti,ab OR "multi sens*".ti,ab OR "multiparametric".ti,ab OR "multiparamet*".ti,ab OR "multi parametric".ti,ab OR "multi paramet*".ti,ab)) OR ((exp *"Heart Failure"/ OR "heart failure".ti,ab OR "Cardio-Renal Syndrome".ti,ab OR "Paroxysmal Dyspnea".ti,ab OR "Cardiac Edema".ti,ab OR "Cardiac Oedema".ti,ab OR "Cardiac Failure".ti,ab OR "Heart Decompensation".ti,ab OR "Myocardial Failure".ti,ab) AND (exp *"Cardiac Resynchronization Therapy"/ OR exp *"Implantable Cardioverter Defibrillators"/ OR "implantable defibrillator".ti,ab OR "implantable cardiac defibrillator".ti,ab OR "implantable cardioverter-defibrillator".ti,ab OR "cardiac implantable electronic device".ti,ab OR "cardiac implanted electronic device".ti,ab OR "implantable device".ti,ab OR "cardiac device".ti,ab OR "implantable cardioverter defibrillator".ti,ab OR "implanted defibrillator".ti,ab OR "cardiac resynchronisation therapy".ti,ab OR "cardiac resynchronization therapy".ti,ab OR "implantable defibrillators".ti,ab OR "implantable cardiac defibrillators".ti,ab OR "implantable cardioverter-defibrillators".ti,ab OR "cardiac implantable electronic devices".ti,ab OR "cardiac implanted electronic devices".ti,ab OR "implantable devices".ti,ab OR "cardiac devices".ti,ab OR "implantable cardioverter defibrillators".ti,ab OR "implanted defibrillators".ti,ab OR "cardiac resynchronisation therap*".ti,ab OR "cardiac resynchronization therap*".ti,ab OR "implantable defibrillator*".ti,ab OR "implantable cardiac defibrillator*".ti,ab OR "implantable cardioverter-defibrillator*".ti,ab OR "cardiac implantable electronic device*".ti,ab OR "cardiac implanted electronic device*".ti,ab OR "implantable device*".ti,ab OR "cardiac device*".ti,ab OR "implantable cardioverter defibrillator*".ti,ab OR "implanted defibrillator*".ti,ab) AND ("multisensor".ti,ab OR "multisens*".ti,ab OR "multi sensor".ti,ab OR "multi sens*".ti,ab OR "multiparametric".ti,ab OR "multiparamet*".ti,ab OR "multi parametric".ti,ab OR "multi paramet*".ti,ab OR "remote*".ti,ab OR "ambulator*".ti,ab OR "tele*".ti,ab OR "monitor*".ti,ab OR exp *"Ambulatory Monitoring"/ OR exp *"Physiologic Monitoring"/) AND ("fluid retention".ti,ab OR exp *"Fluid Retention"/ OR "fluid status".ti,ab OR "fluid index".ti,ab OR "worsening".ti,ab OR "worse*".ti,ab OR "decompensation".ti,ab OR "congestion".ti,ab OR "congestive".ti,ab OR "Flare Up".ti,ab OR "Disease Exacerbation"/)) OR ((exp *"Heart Failure"/ OR "heart failure".ti OR "Cardio-Renal Syndrome".ti OR "Paroxysmal Dyspnea".ti OR "Cardiac Edema".ti OR "Cardiac Oedema".ti OR "Cardiac Failure".ti OR "Heart Decompensation".ti OR "Myocardial Failure".ti) AND (exp *"Cardiac Resynchronization Therapy"/ OR exp *"Implantable Cardioverter Defibrillators"/ OR "implantable defibrillator".ti OR "implantable cardiac defibrillator".ti OR "implantable cardioverter-defibrillator".ti OR "cardiac implantable electronic device".ti OR "cardiac implanted electronic device".ti OR "implantable device".ti OR "cardiac device".ti OR "implantable cardioverter defibrillator".ti OR "implanted defibrillator".ti OR "cardiac resynchronisation therapy".ti OR "cardiac resynchronization therapy".ti OR "implantable defibrillators".ti OR "implantable cardiac defibrillators".ti OR "implantable cardioverter-defibrillators".ti OR "cardiac implantable electronic devices".ti OR "cardiac implanted electronic devices".ti OR "implantable devices".ti OR "cardiac devices".ti OR "implantable cardioverter defibrillators".ti OR "implanted defibrillators".ti OR "cardiac resynchronisation therap*".ti OR "cardiac resynchronization therap*".ti OR "implantable defibrillator*".ti OR "implantable cardiac defibrillator*".ti OR "implantable cardioverter-defibrillator*".ti OR "cardiac implantable electronic device*".ti OR "cardiac implanted electronic device*".ti OR "implantable device*".ti OR "cardiac device*".ti OR "implantable cardioverter defibrillator*".ti OR "implanted defibrillator*".ti) AND ("multisensor".ti,ab OR "multisens*".ti,ab OR "multi sensor".ti,ab OR "multi sens*".ti,ab OR "multiparametric".ti,ab OR "multiparamet*".ti,ab OR "multi parametric".ti,ab OR "multi paramet*".ti,ab OR "remote monitoring".ti,ab OR "ambulatory monitoring".ti,ab OR "telemonitoring".ti,ab OR "monitoring".ti,ab OR "remote monitor*".ti,ab OR "ambulatory monitor*".ti,ab OR "telemonitor*".ti,ab OR (("monitor".ti OR "monitor*".ti OR exp *"Ambulatory Monitoring"/ OR exp *"Physiologic Monitoring"/) AND (exp *"Algorithm"/ OR exp *"Machine Learning"/ OR "Algorithms".ti,ab OR "Algorithm".ti,ab OR "Algorithm*".ti,ab OR "Artificial Intelligence".ti,ab OR "Machine Learning".ti,ab OR "Deep Learning".ti,ab)))) OR (("HeartLogic".ti,ab OR "Heart Logic".ti,ab OR "Triage HF".ti,ab OR "TriageHF".ti,ab OR "HeartInsight".ti,ab))) NOT ((exp "Review"/ OR exp "Systematic Review"/ OR exp "Meta Analysis"/ OR "Case Report"/ OR "case report".ti OR (case AND (report OR reports)).jw.) NOT ("Clinical Study"/ OR "trial".ti OR "RCT".ti)) NOT (exp "Animals"/ NOT exp "Humans"/) AND 2012:2025.(sa_year) |

## Supplemental Table 2: assessment of bias within studies

Assessment of bias within studies included in the meta-analysis using the quality assessment tool ‘QUALSYST’ from the “Standard Quality Assessment Criteria for Evaluating Primary Research Papers from a Variety of Fields” was used. With this tool, 14 items of each quantitative study, were scored on the study and outcome levels depending on the degree to which the specific criteria were met or reported (“yes” = 2, “partial” = 1, “no” = 0). Items not applicable to a particular study design were marked “n/a” and were excluded from the calculation of the summary score.

1. Hindricks G, Taborsky M, Glikson M, Heinrich U, Schumacher B, Katz A, et al. Implant-based multiparameter telemonitoring of patients with heart failure (IN-TIME): a randomised controlled trial. Lancet. 2014;384(9943):583-90.

|  | **CRITERIA** | **YES +2** | **PARTIAL +1** | **NO 0** | **N/A** |
| --- | --- | --- | --- | --- | --- |
| **1** | Question / objective sufficiently described? | x |  |  |  |
| **2** | Study design evident and appropriate? | x |  |  |  |
| **3** | Method of subject/comparison group selection or source of information/input variables described and appropriate? | x |  |  |  |
| **4** | Subject and comparison group (if applicable) characteristics sufficiently described? | x |  |  |  |
| **5** | If interventional and random allocation was possible, was it reported? | x |  |  |  |
| **6** | If interventional and blinding of investigators was possible, was it reported? |  |  |  | x |
| **7** | If interventional and blinding of subjects was possible, was it reported? |  |  |  | x |
| **8** | Outcome and (if applicable) exposure measure(s) well defined and robust to measurement / misclassification bias? Means of assessment reported? | x |  |  |  |
| **9** | Sample size appropriate? | x |  |  |  |
| **10** | Analytic methods described/justified and appropriate? | x |  |  |  |
| **11** | Some estimate of variance is reported for the main results? | x |  |  |  |
| **12** | Controlling for confounding? | x |  |  |  |
| **13** | Results reported in sufficient detail? | x |  |  |  |
| **14** | Conclusion supported by the results? | x |  |  |  |
|  | **TOTAL SCORE** | 24/24 | | | |

1. Boriani G, Da Costa A, Quesada A, Ricci RP, Favale S, Boscolo G, et al. Effects of remote monitoring on clinical outcomes and use of healthcare resources in heart failure patients with biventricular defibrillators: results of the MORE-CARE multicentre randomized controlled trial. Eur J Heart Fail. 2017;19(3):416-25.

|  | **CRITERIA** | **YES +2** | **PARTIAL +1** | **NO 0** | **N/A** |
| --- | --- | --- | --- | --- | --- |
| **1** | Question / objective sufficiently described? | x |  |  |  |
| **2** | Study design evident and appropriate? | x |  |  |  |
| **3** | Method of subject/comparison group selection or source of information/input variables described and appropriate? | x |  |  |  |
| **4** | Subject and comparison group (if applicable) characteristics sufficiently described? | x |  |  |  |
| **5** | If interventional and random allocation was possible, was it reported? | x |  |  |  |
| **6** | If interventional and blinding of investigators was possible, was it reported? |  |  |  | x |
| **7** | If interventional and blinding of subjects was possible, was it reported? |  |  |  | x |
| **8** | Outcome and (if applicable) exposure measure(s) well defined and robust to measurement / misclassification bias? Means of assessment reported? | x |  |  |  |
| **9** | Sample size appropriate? | x |  |  |  |
| **10** | Analytic methods described/justified and appropriate? | x |  |  |  |
| **11** | Some estimate of variance is reported for the main results? | x |  |  |  |
| **12** | Controlling for confounding? | x |  |  |  |
| **13** | Results reported in sufficient detail? | x |  |  |  |
| **14** | Conclusion supported by the results? | x |  |  |  |
|  | **TOTAL SCORE** | 24/24 | | | |

1. Morgan JM, Kitt S, Gill J, McComb JM, Ng GA, Raftery J, et al. Remote management of heart failure using implantable electronic devices. Eur Heart J. 2017;38(30):2352-60.

|  | **CRITERIA** | **YES +2** | **PARTIAL +1** | **NO 0** | **N/A** |
| --- | --- | --- | --- | --- | --- |
| **1** | Question / objective sufficiently described? | x |  |  |  |
| **2** | Study design evident and appropriate? | x |  |  |  |
| **3** | Method of subject/comparison group selection or source of information/input variables described and appropriate? | x |  |  |  |
| **4** | Subject and comparison group (if applicable) characteristics sufficiently described? | x |  |  |  |
| **5** | If interventional and random allocation was possible, was it reported? | x |  |  |  |
| **6** | If interventional and blinding of investigators was possible, was it reported? |  |  |  | x |
| **7** | If interventional and blinding of subjects was possible, was it reported? |  |  |  | x |
| **8** | Outcome and (if applicable) exposure measure(s) well defined and robust to measurement / misclassification bias? Means of assessment reported? | x |  |  |  |
| **9** | Sample size appropriate? | x |  |  |  |
| **10** | Analytic methods described/justified and appropriate? | x |  |  |  |
| **11** | Some estimate of variance is reported for the main results? | x |  |  |  |
| **12** | Controlling for confounding? | x |  |  |  |
| **13** | Results reported in sufficient detail? | x |  |  |  |
| **14** | Conclusion supported by the results? | x |  |  |  |
|  | **TOTAL SCORE** | 24/24 | | | |

1. Bogyi P, Vamos M, Bari Z, Polgar B, Muk B, Nyolczas N, et al. Association of Remote Monitoring With Survival in Heart Failure Patients Undergoing Cardiac Resynchronization Therapy: Retrospective Observational Study. J Med Internet Res. 2019;21(7):e14142.

|  | **CRITERIA** | **YES +2** | **PARTIAL +1** | **NO 0** | **N/A** |
| --- | --- | --- | --- | --- | --- |
| **1** | Question / objective sufficiently described? | x |  |  |  |
| **2** | Study design evident and appropriate? | x |  |  |  |
| **3** | Method of subject/comparison group selection or source of information/input variables described and appropriate? | x |  |  |  |
| **4** | Subject and comparison group (if applicable) characteristics sufficiently described? | x |  |  |  |
| **5** | If interventional and random allocation was possible, was it reported? |  |  |  | x |
| **6** | If interventional and blinding of investigators was possible, was it reported? |  |  |  | x |
| **7** | If interventional and blinding of subjects was possible, was it reported? |  |  |  | x |
| **8** | Outcome and (if applicable) exposure measure(s) well defined and robust to measurement / misclassification bias? Means of assessment reported? |  | x (outcome measures retrospectively assessed in Hungarian National Health Fund Death Registry, which is possibly prone to misclassification bias) |  |  |
| **9** | Sample size appropriate? | x |  |  |  |
| **10** | Analytic methods described/justified and appropriate? | x |  |  |  |
| **11** | Some estimate of variance is reported for the main results? | x |  |  |  |
| **12** | Controlling for confounding? |  | x (adequately adjusted for predefined set of covariates using Cox  proportional hazards regression model, but due to retrospective nature, unmeasured confounders and biases may still be present) |  |  |
| **13** | Results reported in sufficient detail? | x |  |  |  |
| **14** | Conclusion supported by the results? | x |  |  |  |
|  | **TOTAL SCORE** | 20/22 | | | |

1. Ezer P, Farkas N, Szokodi I, Kónyi A. Automatic daily remote monitoring in heart failure patients implanted with a cardiac resynchronisation therapy-defibrillator: a single-centre observational pilot study. Arch Med Sci. 2023;19(1):73-85.

|  | **CRITERIA** | **YES +2** | **PARTIAL +1** | **NO 0** | **N/A** |
| --- | --- | --- | --- | --- | --- |
| **1** | Question / objective sufficiently described? | x |  |  |  |
| **2** | Study design evident and appropriate? | x |  |  |  |
| **3** | Method of subject/comparison group selection or source of information/input variables described and appropriate? | x |  |  |  |
| **4** | Subject and comparison group (if applicable) characteristics sufficiently described? | x |  |  |  |
| **5** | If interventional and random allocation was possible, was it reported? |  |  |  | x |
| **6** | If interventional and blinding of investigators was possible, was it reported? |  |  |  | x |
| **7** | If interventional and blinding of subjects was possible, was it reported? |  |  |  | x |
| **8** | Outcome and (if applicable) exposure measure(s) well defined and robust to measurement / misclassification bias? Means of assessment reported? |  | x (outcome measures retrospectively assessed in integrated patient care information system, which is possibly prone to misclassification bias) |  |  |
| **9** | Sample size appropriate? |  | x (sample size rather small N = 88) |  |  |
| **10** | Analytic methods described/justified and appropriate? | x |  |  |  |
| **11** | Some estimate of variance is reported for the main results? | x |  |  |  |
| **12** | Controlling for confounding? |  | x (Cox  proportional regression was used, but due to retrospective nature, unmeasured confounders and biases may still be present) |  |  |
| **13** | Results reported in sufficient detail? | x |  |  |  |
| **14** | Conclusion supported by the results? | x |  |  |  |
|  | **TOTAL SCORE** | 19/22 | | | |

1. de Juan Bagudá J, Cózar León R, Gavira Gómez JJ, Pachón M, Goirigolzarri Artaza J, Martínez Mateo V, et al. Clinical impact of remote heart failure management using the multiparameter ICD HeartLogic alert. Rev Esp Cardiol (Engl Ed). 2024.

|  | **CRITERIA** | **YES +2** | **PARTIAL +1** | **NO 0** | **N/A** |
| --- | --- | --- | --- | --- | --- |
| **1** | Question / objective sufficiently described? | x |  |  |  |
| **2** | Study design evident and appropriate? | x |  |  |  |
| **3** | Method of subject/comparison group selection or source of information/input variables described and appropriate? | x |  |  |  |
| **4** | Subject and comparison group (if applicable) characteristics sufficiently described? |  | x (retrospective observational study with baseline characteristics reported for the total group, but not separate for HL-ON and HL-OFF period) |  |  |
| **5** | If interventional and random allocation was possible, was it reported? |  |  |  | x |
| **6** | If interventional and blinding of investigators was possible, was it reported? |  |  |  | x |
| **7** | If interventional and blinding of subjects was possible, was it reported? |  |  |  | x |
| **8** | Outcome and (if applicable) exposure measure(s) well defined and robust to measurement / misclassification bias? Means of assessment reported? |  | x (not clearly reported how information of the endpoints was retrieved) |  |  |
| **9** | Sample size appropriate? | x |  |  |  |
| **10** | Analytic methods described/justified and appropriate? | x |  |  |  |
| **11** | Some estimate of variance is reported for the main results? | x |  |  |  |
| **12** | Controlling for confounding? |  | x (due to retrospective nature, unmeasured confounders and biases may still be present) |  |  |
| **13** | Results reported in sufficient detail? | x |  |  |  |
| **14** | Conclusion supported by the results? | x |  |  |  |
|  | **TOTAL SCORE** | 19/22 | | | |

1. Treskes RW, Beles M, Caputo ML, Cordon A, Biundo E, Maes E, et al. Clinical and economic impact of HeartLogic™ compared with standard care in heart failure patients. ESC Heart Fail. 2021;8(2):1541-51.

|  | **CRITERIA** | **YES +2** | **PARTIAL +1** | **NO 0** | **N/A** |
| --- | --- | --- | --- | --- | --- |
| **1** | Question / objective sufficiently described? | x |  |  |  |
| **2** | Study design evident and appropriate? | x |  |  |  |
| **3** | Method of subject/comparison group selection or source of information/input variables described and appropriate? | x |  |  |  |
| **4** | Subject and comparison group (if applicable) characteristics sufficiently described? |  | x (retrospective observational study with baseline characteristics reported for the total group, but not separate for HL-ON and HL-OFF period) |  |  |
| **5** | If interventional and random allocation was possible, was it reported? |  |  |  | x |
| **6** | If interventional and blinding of investigators was possible, was it reported? |  |  |  | x |
| **7** | If interventional and blinding of subjects was possible, was it reported? |  |  |  | x |
| **8** | Outcome and (if applicable) exposure measure(s) well defined and robust to measurement / misclassification bias? Means of assessment reported? |  | x (outcome measures retrospectively assessed in electronic health record, which is possibly prone to misclassification bias) |  |  |
| **9** | Sample size appropriate? |  | x (sample size rather small N = 74) |  |  |
| **10** | Analytic methods described/justified and appropriate? | x |  |  |  |
| **11** | Some estimate of variance is reported for the main results? | x |  |  |  |
| **12** | Controlling for confounding? |  | x (different subanalyses were made, but due to retrospective nature, unmeasured confounders and biases may still be present) |  |  |
| **13** | Results reported in sufficient detail? | x |  |  |  |
| **14** | Conclusion supported by the results? | x |  |  |  |
|  | **TOTAL SCORE** | 18/22 | | | |

1. Feijen M, Beles M, Tan YZ, Cordon A, Dupont M, Treskes RW, et al. Fewer Worsening Heart Failure Events With HeartLogic on top of Standard Care: a Propensity-Matched Cohort Analysis. J Card Fail. 2023.

|  | **CRITERIA** | **YES +2** | **PARTIAL +1** | **NO 0** | **N/A** |
| --- | --- | --- | --- | --- | --- |
| **1** | Question / objective sufficiently described? | x |  |  |  |
| **2** | Study design evident and appropriate? | x |  |  |  |
| **3** | Method of subject/comparison group selection or source of information/input variables described and appropriate? | x |  |  |  |
| **4** | Subject and comparison group (if applicable) characteristics sufficiently described? | x |  |  |  |
| **5** | If interventional and random allocation was possible, was it reported? |  |  |  | x |
| **6** | If interventional and blinding of investigators was possible, was it reported? |  |  |  | x |
| **7** | If interventional and blinding of subjects was possible, was it reported? |  |  |  | x |
| **8** | Outcome and (if applicable) exposure measure(s) well defined and robust to measurement / misclassification bias? Means of assessment reported? |  | x (outcome measures retrospectively assessed in electronic health record, which is possibly prone to misclassification bias) |  |  |
| **9** | Sample size appropriate? | x |  |  |  |
| **10** | Analytic methods described/justified and appropriate? | x |  |  |  |
| **11** | Some estimate of variance is reported for the main results? | x |  |  |  |
| **12** | Controlling for confounding? |  | x (propensity score matching was used, but due to retrospective nature, unmeasured confounders and biases may still be present) |  |  |
| **13** | Results reported in sufficient detail? | x |  |  |  |
| **14** | Conclusion supported by the results? | x |  |  |  |
|  | **TOTAL SCORE** | 20/22 | | | |

1. Ahmed FZ, Sammut-Powell C, Martin GP, Callan P, Cunnington C, Kahn M, et al. Association of a device-based remote management heart failure pathway with outcomes: TriageHF Plus real-world evaluation. ESC Heart Fail. 2024.

|  | **CRITERIA** | **YES +2** | **PARTIAL +1** | **NO 0** | **N/A** |
| --- | --- | --- | --- | --- | --- |
| **1** | Question / objective sufficiently described? | x |  |  |  |
| **2** | Study design evident and appropriate? | x |  |  |  |
| **3** | Method of subject/comparison group selection or source of information/input variables described and appropriate? | x |  |  |  |
| **4** | Subject and comparison group (if applicable) characteristics sufficiently described? | x |  |  |  |
| **5** | If interventional and random allocation was possible, was it reported? |  |  |  | x |
| **6** | If interventional and blinding of investigators was possible, was it reported? |  |  |  | x |
| **7** | If interventional and blinding of subjects was possible, was it reported? |  |  |  | x |
| **8** | Outcome and (if applicable) exposure measure(s) well defined and robust to measurement / misclassification bias? Means of assessment reported? |  | x (outcome measures retrospectively assessed in electronic health record, which is possibly prone to misclassification bias) |  |  |
| **9** | Sample size appropriate? | x |  |  |  |
| **10** | Analytic methods described/justified and appropriate? | x |  |  |  |
| **11** | Some estimate of variance is reported for the main results? | x |  |  |  |
| **12** | Controlling for confounding? |  | x (negative-Binomial Regression with Inverse Probability Treatment Weighting (IPTW) Using a Time-Matched Usual Care Cohort was used, but due to retrospective nature, unmeasured confounders and biases may still be present) |  |  |
| **13** | Results reported in sufficient detail? | x |  |  |  |
| **14** | Conclusion supported by the results? | x |  |  |  |
|  | **TOTAL SCORE** | 20/22 | | | |

1. Landolina M, Perego GB, Lunati M, Curnis A, Guenzati G, Vicentini A, et al. Remote monitoring reduces healthcare use and improves quality of care in heart failure patients with implantable defibrillators: the evolution of management strategies of heart failure patients with implantable defibrillators (EVOLVO) study. Circulation. 2012;125(24):2985-92.

|  | **CRITERIA** | **YES +2** | **PARTIAL +1** | **NO 0** | **N/A** |
| --- | --- | --- | --- | --- | --- |
| **1** | Question / objective sufficiently described? | x |  |  |  |
| **2** | Study design evident and appropriate? | x |  |  |  |
| **3** | Method of subject/comparison group selection or source of information/input variables described and appropriate? | x |  |  |  |
| **4** | Subject and comparison group (if applicable) characteristics sufficiently described? | x |  |  |  |
| **5** | If interventional and random allocation was possible, was it reported? | x |  |  |  |
| **6** | If interventional and blinding of investigators was possible, was it reported? |  |  |  | x |
| **7** | If interventional and blinding of subjects was possible, was it reported? |  |  |  | x |
| **8** | Outcome and (if applicable) exposure measure(s) well defined and robust to measurement / misclassification bias? Means of assessment reported? | x |  |  |  |
| **9** | Sample size appropriate? | x |  |  |  |
| **10** | Analytic methods described/justified and appropriate? | x |  |  |  |
| **11** | Some estimate of variance is reported for the main results? | x |  |  |  |
| **12** | Controlling for confounding? | x |  |  |  |
| **13** | Results reported in sufficient detail? | x |  |  |  |
| **14** | Conclusion supported by the results? | x |  |  |  |
|  | **TOTAL SCORE** | 24/24 | | | |

## Supplemental Table 3: general overview of all included articles

| **First author, journal** | **Type of study** | **Parameter(s) used** | **N** | **Median age (y)** | **Gender (% males)** | **Etiology HF (ICM in %)** | **NYHA class (I/II/III/IV in %)** | **LVEF**  **(in %)** | **FU time in months** | **Intervention** | **Comparator** | **Outcome (primary listed first, secondary thereafter)** | **Result** |
| --- | --- | --- | --- | --- | --- | --- | --- | --- | --- | --- | --- | --- | --- |
| Landolina M.  *Circulation*  *2012*  *EVOLVO study* | Prospective, multicenter, randomised | - AT/AF burden  - V rate during AT/AF  - ICD therapy  - Thoracic impedance | 200 | 66 ± 6 | 82 | 38 | 11/72/17 (I+II+III) | 31 (25-35) | 16 - 0 | Remote | Standard FU | 1.Rate of ED or urgent visits for HF, arrhythmias or ICD-events  2.Rate of ED or urgent visits due to WHF  3.Rate of ED or urgent visits due to arrhythmias or ICD-events | 1.Rate of ED or urgent visits for HF, arrhythmias or ICD-events  *IRR 0.65 (0.49 – 0.88, P 0.005)*  2.Rate of ED or urgent visits due to WHF  *IRR 0.52 (0.37 – 0.75, P < 0.001)*  3.Rate of ED or urgent visits due to arrhythmias or ICD-events  *IRR 1.14 (0.65 – 1.99, P 0.649)* |
| Cowie M.  *EHJ*  *2013* | Retrospective, multicenter | Triage HF parameters | 1310 | 67 ± 11 | 74 | 61 | 4/22/70/4 | 92% of patients with LVEF <35% | 11 ± 6 | Low HFRS  Intermediate HFRS  High HFRS | N/A | HF hospitalisation | HF hospitalisation (compared to low risk)  *Medium risk: HR 2.1 (1.3 - 3.4, P 0.001)*  *High risk: HR 10.0 (6.4 - 15.7, P < 0.001)* |
| Gula L.  *Heart Rhythm*  *2014* | Substudy RAFT trial which was multicenter, prospective, randomised | Triage HF parameters | 1224 | 66 ± 9 | 83 | 65 | 0/87/13/0 | 23 ± 5 | 31 – no SD | Low HFRS  Intermediate HFRS  High HFRS | N/A | HF hospitalisation | HF hospitalisation (*compared to low risk)*  *Medium risk: RR 2.9 (2.0 - 4.4)*  *High risk: RR 10.7 (6.9 - 16.6)* |
| Hindricks G.  *Lancet*  *2014*  *IN TIME study* | Prospective, multicenter, randomised | -Onset of arrhythmias  -Duration of daily physical activity  -Ventricular ectopy (VES/h)  -Heart rate variability  Mean heart rate (24 h)  -Mean heart rate at rest | 664 | 66 ± 9 | 81 | 69 | 0/43/57/0 (II+III) | 26 ± 7 | 11 ± 3 | Remote | Standard FU | 1.Composite (all-cause death, overnight hospital admission for heart failure, change in NYHA class, and change in patient global self-assessment)  2. All cause death  3. Admission for WHF | 1.Composite  *18.9% vs. 27.2% (P 0.013)*  2.All cause death  *3.0% vs. 8.2% (P 0.004)*  3.Admission for WHF  *6.9% vs. 8.2%* |
| Sharma V.  *Heart Lung*  *2015* | Retrospective, mutlicenter | -Patient activity  -Day and night HR  -AT/AF burden  - V rate during afib  -% BiV pacing  -ICD therapies  -Thoracic impedance | 775 | 69 ± 11 | 68 | 63 | 9/59/674/33 | 100% of patients with LVEF<35 | 13 ± 5 | 0,1,2,3 device observations | N/A | Heart failure hospitalisation | Heart failure hospitalisation (vs. 0 device observations)  *1 device observation*  *OR 4.6 (1.4 - 14.5)*  *2 device observations*  *OR 14,9 (5.2 - 43.1)*  *3 device observations*  *OR 42.4 (12.6 - 142.1)* |
| Brasca F.  *J Telemed Telecare*  *2016* | Retrospective, single center | -AT/AF burden  -V rate during Afib  -Thoracic impedance  -Pt activity  -HRV  -Nocturnal HR  -% BiV pacing  -ICD therapy | 104 | 70 ± 8 | 81 | 51 | 48% (II) | 29 ± 6 | 21 (range: 1-67) | PARTNERS HF score >= 2 | N/A | HF events (=hospitalisation or unplanned HF visit due to congestion) | HF events (=hospitalisation or unplanned HF visit due to congestion)  *Sensitivity 75%, specificity 68%*  *OR 6.24 (4.90 - 7.95, P < 0.001)* |
| Boehmer J.  *JACC HF*  *2017*  *MultiSENSE study* | Prospective, non-randomized, multicenter | HeartLogic parameters | 400 | 67 ± 10 | 72 | 49 | 5/69/25/1 | 30 ± 11 | 11 (0.59-12) | HL ‘IN’ alert | N/A | HF events (= admissions or unscheduled visits with IV treatment) | HF events (= admissions or unscheduled visits with IV treatment)  *Sensitivity 70%* |
| Boriani G.  *EHJ HF*  *2017*  *MORE CARE trial* | Prospective, randomized, multicenter | -Thoracic impedance  -AT/AF burden  -System integrity | 865 | 66 ± 11 | 79 | 43 | 63% (III+IV) | 27 ± 7 | 25 (14-26) | Remote | In office FU | 1.All cause mortality, CV and device-related hospitalisation  2.All cause mortality  3.CV hospitalisation  4.Device related hospitalisation  5. HF hospitalisation  6. Outpatient visits | 1.All cause mortality, CV and device-related hospitalisation  *HR 1.02 (0.80 - 1.30, P 0.889)*  2.All cause mortality  *HR 1.13 (0.71-1.80, P 0.594)*  3.CV hospitalisation  *HR 0.96 (0.73-1.28, P 0.796)*  4.Device related hospitalisation  *HR 0.89 (0.44-1.79, P 0.742)*  5. HF hospitalisation  *IRR 0.97 (0.74 - 1.29, P 0.846)*  6. Outpatient visits  *IRR 0.59 (0.56 - 0.62, P < 0.001)* |
| Morgan J.  *EHJ*  *2017*  *REM HF trial* | Multicenter, randomised, prospective | -%BiV pacing  -Nocturnal HR  -Thoracic impedance  -Patientt activity  -AT/AF burden  -Ventricular arrhythmias  -ICD therapy  -HRV  -Lead integrity  -Device programming  -V-V interval | 1650 | 70 ± 10 | 86 | 60 | 0/70/30/0 | 30 ± 10 | 34 (range: 0-52) | Remote | Usual care | 1. All cause mortality + CV hospitalisation  2.All cause mortality  3.CV mortality  4.CV hospitalisation | 1. All cause mortality + CV hospitalisation  *HR 1.01 (0.87 - 1.18, P 0.8727)*  2.All cause mortality  *HR 0.83 (0.66 - 1.05, P 0.1231)*  3.CV mortality  *HR 0.88 (0.68 - 1.14, P 0.3387)*  4.CV hospitalisation  *HR 1.07 (0.91 - 1.25, P 0.4195)* |
| Burri H.  *Europace*  *2018*  *MORE CARE substudy* | Post hoc analysis of a prospective, randomised, multicenter trial | TriageHF parameters | 722 | 66 ± 10 | 76 | 44 | 7/32/58/3 | 27 ± 6 | 20 (11-23) | Low HFRS  Intermediate HFRS  High HFRS | N/A | 1.CV hospitalisation  2.HF hospitalisation  3.Non HF CV hospitalisation | 1.CV hospitalisation  *High vs Low: RR 4.5 (3.1-6.6, P < 0.001)*  2.HF hospitalisation  *High vs Low RR 6.3 (3.9-10.2, P < 0.001)*  3.Non HF CV hospitalisation  *High vs Low RR 3.5 (2.0-6.0, P < 0.001)* |
| Gardner R.  *Circ Heart Fail*  *2018*  *MULTISENSE substudy* | Prospective, non-randomized, multicenter | HeartLogic parameters | 900 | 67 ± 11 | 73 | 51 | 5/67/27/<1 | 30 ± 11 | 13 (13-13) | HL ‘IN’ alert | HL ‘OUT’ alert | HF events (= combination of death, hospitalisation and outpatient visit with IV/PO treatment) | HF events (= combination of death, hospitalisation and outpatient visit with IV/PO treatment)  *ERR 7.05 (4.69 - 10.61, P < 0.0001)* |
| Bogyi P.  *J Med Internet Res*  *2019* | Retrospective, single-center | -Thoracic impedance  -AT/AF burden  -V rate during afib  -ICD therapy  -lead/device integrity  -Battery life | 231 | 64 ± 10 | 82 | 53 | Mean ± SD:  2.4 ± 0.7 | 27 ± 7 | 28 ± 18 | Remote monitoring | Conventional FU | All cause mortality | All cause mortality  *HR 0.368 (0.186-0.727, P = 0.004)* |
| Okumura K.  *Circ J*  *2020*  *SCAN HF study* | Prospective, non-randomized, multi-center | TriageHF parameters | 315 | 68 ± 12 | 70 | 24 | 6/52/41/1 | 31 ± 11 | 22 ± 12 | Low HFRS  Intermediate HFRS  High HFRS | N/A | HF hospitalisation | HF hospitalisation  *Medium to low: RR 2.18 (1.23 - 3.85)*  *High to low: RR 5.78 (3.34-10.01)* |
| Zile M.  *ESC Heart Failure*  *2020* | Retrospective,  multicenter | TriageHF parameters | 22 901 | 66 ± 12 | 71 | - | - | - | 22 ± 16 | Low HFRS  Intermediate HFRS  High HFRS | N/A | 1.HF hospitalisation  2.All cause mortality | 1.HF hospitalisation  *Low vs intermediate: OR 2.8 (2.5-3.2, P < 0.001)*  *Intermediate vs high: OR 9.2 (8.1-10.3, P < 0.001)*  2.All cause mortality  *Low vs intermediate: aHR 1.8 (1.4-2.2, P < 0.001)*  *Low vs high: aHR 3.5 (2.8-4.3, P < 0.001)* |
| Calo L.  *Circ Heart Fail*  *2021* | Prospective, non-randomized, multicenter | HeartLogic parameters | 366 | 69 ± 11 | 78 | 47 | 7/54/37/2 | 31 ± 9 | 11 (6-16) | HL ‘IN’ alert | HL ‘OUT’ alert | 1.HF hospitalisation and HF death  2.Time to HF death | 1.HF hospitalisation and HF death  *HR 30.63 (13.04 - 71.95) P < 0.001*  2.Time to HF death  *HR 11.45 (5.55 - 23.60) P < 0.001* |
| Gardner R.  *ESC Heart Failure*  *2021*  *MULTISENSE substudy* | Prospective, non-randomized, multicenter | HeartLogic parameters | 900 | 67 ± 11 | 73 | 51 | 5/67/27/<1 | 30 ± 11 | 13 (13-13) | HL ‘IN’ alert | N/A | HL score between patients in clinically stable periods without any HF event vs. with HF event during study FU | HL score between patients in clinically stable periods without any HF event vs. with HF event during study FU  5.31 vs. 11.07 (P 0.001) |
| Treskes R.  *ESC Heart Failure*  *2021* | Prospective, non-randomized, multicenter | HeartLogic parameters | 74 | 67 ± 10 | 84 | 36 | 20/47/32/0 | 31 ± 11 | 12 (0) | HF events after activation | HF events before activation | 1.HF hospitalisations  2.Length of stay | 1.HF hospitalisations  *27 vs. 7 (P 0.003)*  2.Length of stay  *16 vs. 7 (P 0.079)* |
| Ahmed F.  *Europace*  *2022* | Prospective, non-randomized, single center | TriageHF parameters | 439 | 66 ± 16 | 63 | 56 | 13/34/34/5 – 14% without HF | 56% of patients with LVEF<35 | 23 (13-28) | High HFRS (at the start or during FU) | Never high HFRS | All cause mortality | All cause mortality  *OR 3.07 (1.57 - 6.58, P = 0.002)* |
| Baguda J.  *Rev Esp Cardiol*  *2022*  *RE-HEART registry* | Prospective, non-randomized, multicenter | HeartLogic parameters | 288 | 68 ± 10 | 77 | 52 | 16/58/26/- | 75% of patients with LVEF <35% | Phase 1:  10 (5-19) Phase 2+3: 16 (15-22) | HL ‘IN’ alert | N/A | HF events and death (hospitalization/unscheduled visit requiring intravenous treatment or resulting in HF death) | HF events and death (hospitalization/unscheduled visit requiring intravenous treatment or resulting in HF death)  *Sensitivity 98%, specificity 90%*  *PPV 29%, NPV 99.9%* |
| D’Onofrio A.  *Europace*  *2022*  *SELENE HF study* | Prospective, non-randomized, multicenter | HeartInsight parameters | 461 | 69 ± 8 | 82 | 45 | 0/48/52/0 | 30 (25-35) | 23 (15-37) | Nominal threshold 4.5 | N/A | 1.HF hospitalisation  2.Composite of any hospitalisation, outpatient IV diuretics or HF death | For nominal threshold of 4.5  1.HF hospitalisation  *Sensitivity 65.5%, specificity 86.7%*  *False-alert 0.69 alerts per patient-year*  2.Composite of any hospitalisation, outpatient IV diuretics or HF death  *Sensitivity 54.8%, specificity 86.5%*  *False-alert 0.67 alerts per patient-year* |
| Samut-Powell C.  *J Am Heart Assoc*  *2022* | Prospective, non-randomized, single center | TriageHF parameters | 435 | 66 ± 16 | 63 | - | 13/35/33 (III+IV) – 14% without HF | 56% of patients with LVEF<35 | 17 – no SD | ‘High’  (at baseline, or during FU) | Non ‘high’ | 1.All-cause hospitalisation  2.CV hospitalisation  3.HF hospitalisation | 1.All-cause hospitalisation  *Sensitivity 37.3%, specificity 86.2%*  *HR 2.9 (95% CI, 1.88–­4.40; P<0.001)*  2.CV hospitalisation  *Sensitivity 39.3%, specificity 85.7%*  *HR 4.1 (95% CI, 2.08–­8.01; P<0.001)*  3.HF hospitalisation  Sensitivity 62.5%, specificity 85.6%  *HR not shown* |
| Boriani G.  *Europace*  *2023* | Retrospective,  multicenter | HeartLogic parameters | 568 | 69 ± 10 | 80 | 50 | 6/62/30/2 | 32 ± 9 | 26 (16-37) | HL ‘IN’ alert in sinus rhythm or afib | HL ‘OUT’ alert in sinus rhythm or afib | HF hospitalisations | HF hospitalisations  *SR: IRR 8.59 (1.67 - 55.31)*  *AFIB: IRR 2.70 (1.01 - 28.33)* |
| Cardoso I.  *J Innov Cardiac Rhythm Manage*  *2023* | Retrospective, single-center | TriageHF parameters | 40 | 72 ± 10 | - | 38 | 0/48/48/5 | 27 ± 8 | 36 ± 11 | Low HFRS  Intermediate HFRS  High HFRS | N/A | HF hospitalisations | HF hospitalisations  *Stepwise increase HFRS: OR 12.7 (3.2 - 51.5) P < 0.001* |
| D’Onofrio A.  *Heart Rhythm*  *2023* | Prospective, non randomised, multicenter | HeartLogic parameters | 568 | 69 ± 10 | 80 | 50 | 6/62/30/2 | 32 ± 9 | 26 (16-37) | HL ‘IN’ alert | HL ‘OUT’ alert | All cause mortality | All cause mortality  *IRR 13.72 (7.62 - 25.60, P < 0.001)* |
| Ezer P.  *Arch Med Sci*  *2023* | Retrospective, single-center | -Current rhythm  -Pacemaker dependency  -Mean V rate  -Battery life expectancy  -Lead impedance  -Pacing thresholds  -Sensing signal amplitude  -Arrhythmia events  -ICD Therapy given  -%BiV pacing -Inappropriate shocks  -Dive-triggered alert events  -Patient activity level  -HRV  -Intrathoracic impedance | 88 | 61 ± 11 | 78 | 57 | 0/27/60/13 | 30 ± 5 | 25 | Remote monitoring | Conventional ambulatory FU | 1.CV mortality  2.CV hospitalisations  3.HF hospitalisations  4.Ambulatory visits | 1.CV mortality  *1 vs. 6 (P 0.004)*  2.CV hospitalisations  *37 vs. 46 (P 0.076)*  3.HF hospitalisations  *8 vs. 29 (P 0.046)*  4.Ambulatory visits  *6 vs. 19 (P 0.012)* |
| Feijen M.  *J Card Fail*  *2023* | Retrospective, multicenter | HeartLogic parameters | 127 | 68 (59-76) | 80 | 46 | III+IV: 30% | 35 (27-45) | 12 (0) | HeartLogic | Regular TM | 1.Worsening HF events (hospitalisation or unplanned ambulatory visit with treatment modification because of congestion)  2.HF hospitalisations  3.Duration of hospitalisation  4.Ambulatory visits | 1.Worsening HF events  *2 vs. 1 (P 0.004)*  2.HF hospitalisations  *17 vs. 8 (P 0.096)*  3.Duration of hospitalisation  *8 vs. 5 (P 0.023)*  4.Ambulatory visits  *2 vs. 1 (P 0.0001)* |
| Santobuono V.  *ESC Heart Failure*  *2023* | Prospective, non-randomized, multicenter | HeartLogic parameters | 568 | 69 ± 10 | 80 | 50 | 6/62/30/2 | 32 ± 9 | 26 (16-37) | HeartLogic ‘IN’ alert | HeartLogic ‘OUT’ alert | 1.CV hospitalisations  2.CV hospitalisations + death | Incidence rate ratio (IRR)   1. CV hospitalisations   *12.98 (7.16–24.35) P < 0.001*   1. CV hospitalisations + death   *13.35 (8.83–20.51) P < 0.001* |
| Ahmed F.  *ESC Heart Failure*  *2024* | Prospective, non-randomized, multicenter | TriageHF parameters | 758 | 68 ± 13 | 72 | 58 | 20/43/28 (III+IV) | 35% of patients with LVEF<35 | 14 ± 4 | TriageHF | Usual care | 1. Non-elective hospitalizations (all-cause, CV and HF) | 1. Non-elective hospitalizations (all-cause, CV and HF)  IRR 0.42 (0.23-0.76, P = 0.004) |
| Singh J.  *Journal of Cardiac Failure*  *2024* | Retrospective, multicenter | HeartLogic parameters | 1458 | 74 ± 8 | 72 | 81 | - | - | - | HeartLogic | N/A | 1. HF event (acute inpatient event with HF as primary diagnosis, or primary outpatient HF visit with IV diuretics) | HF event (acute inpatient event with HF as primary diagnosis, or primary outpatient HF visit with IV diuretics)   - Sensitivity 74,5% |
| Baguda J.  *Rev Esp Cardiol*  *2024*  *RE-HEART registry* | Prospective, non-randomized, multicenter | HeartLogic parameters | 392 | 69 ± 10 | 76 | 50 | 16/59/25/0 | 71% of patients with LVEF<35 | 17 (10-27) | HeartLogic after activation | HeartLogic before activation | 1. HF hospitalization, or all-cause death  2. Unplanned outpatient HF visit | 1. HF hospitalization, or all-cause death  11% vs 22% (P < 0.001)  2. Unplanned outpatient HF visit  12% vs 24% (P < 0.001) |

**Supplemental table 2:** overview of included articles including the first author, journal, year of publication, type of study, parameter(s) used, number of included patients (N), median age, gender, etiology of heart failure, NYHA class, mean left ventricular ejection fraction (LVEF), follow-up duration, intervention, comparator (if applicable), main outcomes (primary and/or secondary), and results. Whenever the baseline characteristics of the study and intervention groups were published separately, the mean value of the two groups combined was represented. Afib: atrial fibrillation. aHR: adjusted hazard ratio. AT/AF: atrial tachycardia/atrial fibrillation. BiV: biventricular. CV: cardiovascular. ED: emergency department. ERR: event rate ratio. FU: follow-up. HF: heart failure. HFRS: heart failure risk score. HL: HeartLogic. HR: hazard ratio. HRV: heart rate variability. ICD: implantable cardioverter defibrillator. ICM: ischemic cardiomyopathy. IRR: incidence rate ratio. IV: intravenous. NPV: negative predictive value. OR: odds ratio. PO: peroral. PPV: positive predictive value. RR: relative risk. TM: telemonitoring. V: ventricular. VES: ventricular extrasystole. WHF: worsening heart failure.

## Supplemental Table 4: interventions to device-based alerts

| **Clinical reaction to remotely monitored HF alerts** | |
| --- | --- |
| Landolina et al 2012 | Telephone contact with assessment of   - symptoms of decompensation - weight increase - medical therapy adherence - diet adherence - other causes of impedance decrease   Based upon this information:   - Adjust medical therapy if needed - Schedule weekly patient transmission until episode termination - Schedule extra in office visit if needed |
| Hindricks et al 2014 | Telephone contact to assess if there are symptoms suggestive for decompensation. The clinical response to telemonitoring observations was done at the discretion of investigators. |
| Boriani et al 2017 | Telephone contact with assessment of   - symptoms of decompensation - weight increase - medical therapy adherence - diet adherence - other causes of impedance decrease   Based upon this information:   - Adjust medical therapy if needed - Schedule weekly patient transmission until episode termination - Schedule extra in office visit if needed |
| Morgan et al 2017 | Remote monitoring clinical management procedures were standardized across centres and formalized in a Procedural Handbook that guided reactions to active RM changes – options were:   - to do nothing other than review the situation remotely within a short space of time, - reinforce lifestyle advice (e.g. diet, salt intake, exercise), - make changes to medication (such as increase in diuretic dosage) - encourage compliance with medication, - trigger a clinical review by the primary or secondary care team. |
| Bogyi et al 2019 | If a clinically relevant event was perceived, patients were contacted via phone calls and were invited to the clinic for a personal visit in a week. |
| Treskes et al 2021 | Telephone contact with patient if HL index > 16  - If 2 or more symptoms/signs of HF according to ESC guidelines: lifestyle advice and/or treatment adjustment at the discretion of the treating physician  - If less than 2 symptoms/signs of HF: new telephone contact after 2 weeks |
| Ezer et al 2023 | In the presence of one major alert predictor for a HF event: telephone contact to schedule an in-office visit.  Defined major alert were:  - sustained ventricular arrhythmia  - (in)appropriate ICD therapy (shock and/or ATP)  - AT/AF burden > 6h  In the presence of two minor alert predictors for a HF event: telephone contact to assess symptoms. Only if symptoms present: in-office visit.  Defined minor alerts were:  - Decrease in thoracic impedance of 20% in the past week  - BiV pacing perecentage below 90% in the past week  - Decrease of patient activity (< 1h per day) in the past week  - Decrease in HRV (< 60ms) in the past week  - Increase resting HR (> 90/min) in the past week |
| Feijen et al 2023 | Telephone contact with patient if HL index > 16  - If 2 or more symptoms/signs of HF according to ESC guidelines: lifestyle advice and/or treatment adjustment at the discretion of the treating physician  First step: double the dose of oral loop diuretics for 3d with new telephone contact afterwards  - no more symptoms/signs: loop diuretics again to baseline dose  - persisting symptoms/signs: continuation of the elevation loop diuretic dose and/or scheduling for IV treatment  - If less than 2 symptoms/signs of HF: new telephone contact after 2 weeks |
| Baguda et al 2024 | Weekly remote revision of HL-status  If HL-index > 16 : no medical action, but control after 1 week  - Alarm has ended: continue with routine revisions  - Alarm persists: telephone contact  - Clinical worsening: actions at the discretion of the treating physician  - No clinical worsening: patient education and reevaluation after 2 to 6 weeks |
| Ahmed et al 2024 | High TriageHF alert: structured telephone contact  Medium TriageHF alert: review the alert status after 21 days  Low TriageHF alert: routine care  Clinical actions were at the discretion of the care team, including changes to medication, investigations, and advice on lifestyle improvements |

**Supplemental Table 3**: overview of the interventions made to device-based alerts in each of the included studies.

## Supplemental Table 5: PRISMA 2020 checklist

| **Section and Topic** | **Item #** | **Checklist item** | **Location where item is reported** |
| --- | --- | --- | --- |
| **TITLE** | | |  |
| Title | 1 | Identify the report as a systematic review. | Title page |
| **ABSTRACT** | | |  |
| Abstract | 2 | See the PRISMA 2020 for Abstracts checklist. | See Suppl Table 6 |
| **INTRODUCTION** | | |  |
| Rationale | 3 | Describe the rationale for the review in the context of existing knowledge. | P 4-6 |
| Objectives | 4 | Provide an explicit statement of the objective(s) or question(s) the review addresses. | P 4-6 |
| **METHODS** | | |  |
| Eligibility criteria | 5 | Specify the inclusion and exclusion criteria for the review and how studies were grouped for the syntheses. | P 7 |
| Information sources | 6 | Specify all databases, registers, websites, organisations, reference lists and other sources searched or consulted to identify studies. Specify the date when each source was last searched or consulted. | P 6-7 |
| Search strategy | 7 | Present the full search strategies for all databases, registers and websites, including any filters and limits used. | P7, Suppl Table 1 |
| Selection process | 8 | Specify the methods used to decide whether a study met the inclusion criteria of the review, including how many reviewers screened each record and each report retrieved, whether they worked independently, and if applicable, details of automation tools used in the process. | P 7-8 |
| Data collection process | 9 | Specify the methods used to collect data from reports, including how many reviewers collected data from each report, whether they worked independently, any processes for obtaining or confirming data from study investigators, and if applicable, details of automation tools used in the process. | P 7-8 |
| Data items | 10a | List and define all outcomes for which data were sought. Specify whether all results that were compatible with each outcome domain in each study were sought (e.g. for all measures, time points, analyses), and if not, the methods used to decide which results to collect. | P 7-8 |
|  | 10b | List and define all other variables for which data were sought (e.g. participant and intervention characteristics, funding sources). Describe any assumptions made about any missing or unclear information. | P 7-8 |
| Study risk of bias assessment | 11 | Specify the methods used to assess risk of bias in the included studies, including details of the tool(s) used, how many reviewers assessed each study and whether they worked independently, and if applicable, details of automation tools used in the process. | P 7-8, Suppl Table 2 |
| Effect measures | 12 | Specify for each outcome the effect measure(s) (e.g. risk ratio, mean difference) used in the synthesis or presentation of results. | P 8 |
| Synthesis methods | 13a | Describe the processes used to decide which studies were eligible for each synthesis (e.g. tabulating the study intervention characteristics and comparing against the planned groups for each synthesis (item #5)). | P 7-8 |
|  | 13b | Describe any methods required to prepare the data for presentation or synthesis, such as handling of missing summary statistics, or data conversions. | P 8 |
|  | 13c | Describe any methods used to tabulate or visually display results of individual studies and syntheses. | P 8 |
|  | 13d | Describe any methods used to synthesize results and provide a rationale for the choice(s). If meta-analysis was performed, describe the model(s), method(s) to identify the presence and extent of statistical heterogeneity, and software package(s) used. | P 8 |
|  | 13e | Describe any methods used to explore possible causes of heterogeneity among study results (e.g. subgroup analysis, meta-regression). | P 8 |
|  | 13f | Describe any sensitivity analyses conducted to assess robustness of the synthesized results. | P 8 |
| Reporting bias assessment | 14 | Describe any methods used to assess risk of bias due to missing results in a synthesis (arising from reporting biases). | N/A |
| Certainty assessment | 15 | Describe any methods used to assess certainty (or confidence) in the body of evidence for an outcome. | P 8 |
| **RESULTS** | | |  |
| Study selection | 16a | Describe the results of the search and selection process, from the number of records identified in the search to the number of studies included in the review, ideally using a flow diagram. | P 8-9 |
|  | 16b | Cite studies that might appear to meet the inclusion criteria, but which were excluded, and explain why they were excluded. | P 8-16 |
| Study characteristics | 17 | Cite each included study and present its characteristics. | P 8-16 |
| Risk of bias in studies | 18 | Present assessments of risk of bias for each included study. | Suppl Table 2 |
| Results of individual studies | 19 | For all outcomes, present, for each study: (a) summary statistics for each group (where appropriate) and (b) an effect estimate and its precision (e.g. confidence/credible interval), ideally using structured tables or plots. | Figure 3 |
| Results of syntheses | 20a | For each synthesis, briefly summarise the characteristics and risk of bias among contributing studies. | P 8-16, Suppl Table 2, Figure 3 |
|  | 20b | Present results of all statistical syntheses conducted. If meta-analysis was done, present for each the summary estimate and its precision (e.g. confidence/credible interval) and measures of statistical heterogeneity. If comparing groups, describe the direction of the effect. | P 8-16, Figure 3 |
|  | 20c | Present results of all investigations of possible causes of heterogeneity among study results. | P 8-16, Figure 3 |
|  | 20d | Present results of all sensitivity analyses conducted to assess the robustness of the synthesized results. | Figure 3 |
| Reporting biases | 21 | Present assessments of risk of bias due to missing results (arising from reporting biases) for each synthesis assessed. | N/A |
| Certainty of evidence | 22 | Present assessments of certainty (or confidence) in the body of evidence for each outcome assessed. | Figure 3 |
| **DISCUSSION** | | |  |
| Discussion | 23a | Provide a general interpretation of the results in the context of other evidence. | P 16-18 |
|  | 23b | Discuss any limitations of the evidence included in the review. | P 16-18 |
|  | 23c | Discuss any limitations of the review processes used. | P 18 |
|  | 23d | Discuss implications of the results for practice, policy, and future research. | P 16-18 |
| **OTHER INFORMATION** | | |  |
| Registration and protocol | 24a | Provide registration information for the review, including register name and registration number, or state that the review was not registered. | P 7 |
|  | 24b | Indicate where the review protocol can be accessed, or state that a protocol was not prepared. | P 7 |
|  | 24c | Describe and explain any amendments to information provided at registration or in the protocol. | N/A |
| Support | 25 | Describe sources of financial or non-financial support for the review, and the role of the funders or sponsors in the review. | COI page, P 2 |
| Competing interests | 26 | Declare any competing interests of review authors. | COI page, P 2 |
| Availability of data, code and other materials | 27 | Report which of the following are publicly available and where they can be found: template data collection forms; data extracted from included studies; data used for all analyses; analytic code; any other materials used in the review. | P 7 |

## Supplemental Table 6: PRIMSA 2020 checklist for abstracts

| **Section and Topic** | **Item #** | **Checklist item** | **Reported (Yes/No)** |
| --- | --- | --- | --- |
| **TITLE** | | |  |
| Title | 1 | Identify the report as a systematic review. | **DONE** |
| **BACKGROUND** | | |  |
| Objectives | 2 | Provide an explicit statement of the main objective(s) or question(s) the review addresses. | **DONE** |
| **METHODS** | | |  |
| Eligibility criteria | 3 | Specify the inclusion and exclusion criteria for the review. | **DONE** |
| Information sources | 4 | Specify the information sources (e.g. databases, registers) used to identify studies and the date when each was last searched. | **DONE** |
| Risk of bias | 5 | Specify the methods used to assess risk of bias in the included studies. | **N/A due to word limit**  Available in the full text |
| Synthesis of results | 6 | Specify the methods used to present and synthesise results. | **N/A due to word limit**  Available in the full text |
| **RESULTS** | | |  |
| Included studies | 7 | Give the total number of included studies and participants and summarise relevant characteristics of studies. | **DONE** |
| Synthesis of results | 8 | Present results for main outcomes, preferably indicating the number of included studies and participants for each. If meta-analysis was done, report the summary estimate and confidence/credible interval. If comparing groups, indicate the direction of the effect (i.e. which group is favoured). | **DONE** |
| **DISCUSSION** | | |  |
| Limitations of evidence | 9 | Provide a brief summary of the limitations of the evidence included in the review (e.g. study risk of bias, inconsistency and imprecision). | **DONE** |
| Interpretation | 10 | Provide a general interpretation of the results and important implications. | **DONE** |
| **OTHER** | | |  |
| Funding | 11 | Specify the primary source of funding for the review. | **N/A due to word limit**  Available in the full text |
| Registration | 12 | Provide the register name and registration number. | **N/A due to word limit**  Available in the full text |
